# Ascending needles in a haystack? The heterogeneous political participation effects of associational involvement by education

**DOI:** 10.3389/fsoc.2025.1584885

**Published:** 2025-08-07

**Authors:** Sinisa Hadziabdic

**Affiliations:** Max Planck Institute for the Study of Societies, Cologne, Germany

**Keywords:** voluntary associations, political participation, education, civil society, schools of democracy, standard socioeconomic model, panel data

## Abstract

Relying on the data of the Swiss Household Panel, the paper examines the extent to which active involvement in different types of voluntary associations can bridge the political participation gap between individuals with different levels of education. Descriptive analyses reveal that individuals with higher levels of education are significantly more likely to be involved in all types of associations (expressive, instrumental, advocacy). The same education gap holds for all forms of political engagement (attitudinal, institutional, protest, community-oriented, individual). While education acts as a gateway mechanism that affects the likelihood of becoming affiliated with an association, the propensity to actively engage in associational activities after joining is largely independent of education. A longitudinal approach controlling for time-invariant endogeneity shows that active associational involvement increases almost all forms of political engagement. Due to their lower political participation prior to joining, these positive effects are in most cases more pronounced for individuals with low qualifications. Thus, most types of associations effectively reduce the educational gap in political participation. This is particularly true for those forms of participation that are most congruent with associational activities, such as membership in political parties and community-based voluntary work. Political engagement gains are higher among highly educated members only under certain circumstances. This happens either in associational contexts where internal responsibilities are skewed in their favor, as in the case of environmental and charitable organizations, or where the raison d’être of the association implies homogeneous educational qualifications, as in the case of exclusive sports clubs and specific trade unions. Individualized forms of political participation, such as boycotts, are the forms of engagement least affected by the collective action that takes place in associations. Taken together, these empirical findings suggest that associations are an often neglected channel of social mobility, mitigating political differences between social classes and thus promoting social cohesion. At the same time, they offer a more nuanced view, emphasizing that not all associations are capable of bridging the political capital of different social classes and that not all political attitudes are malleable enough to be modified.

## 1 Introduction: stratified “schools of democracy”?

The essence of democratic functioning is based on the idea that the equity of political outcomes is a direct consequence of the fact that all citizens affected by them have actively and equally participated in their formation. However, formal political freedom and equality are meaningless if they are divorced from the actual ability to exercise them (Marx, 1990). The formal right to be politically active is worthless if one does not have the time and knowledge to learn how to become politically influential. Since the early stages of democracy, involvement in voluntary associations has been described as a way to overcome differences between social classes in their ability to participate in political life (de Tocqueville, 2016). By involving their members in activities that are often homologous to those of the democratic process, the knowledge, skills, and attitudes acquired in associations can be directly transferred to the political sphere, thereby increasing the political participation of citizens from disadvantaged segments of society. For this reason, voluntary associations are often portrayed as “schools of democracy” that bridge inequalities between members from different social strata (e.g., Verba et al., 1995; Putnam, 2000).

While there is an extensive literature on whether and how associations can act as democratic equalizers, most of these studies suffer from a number of limitations. We can divide them into two main categories: assuming rather than testing the homogeneity of the relationship between associational involvement and political participation, and inadequately addressing the endogeneity issues that bias the relationship. Associations are often treated as homogeneous contexts that all have similar effects on political participation (e.g., van Ingen and van der Meer, 2016), whereas in reality there are strong differences between different types of associations in terms of their internal activities and thus their qualitatively and quantitatively different ability to influence political participation (e.g., van der Meer and van Ingen, 2009; Binder, 2021). Even when distinguishing between different types of associations, it is often assumed that members of associations have the same experiences within them (e.g., Kerrissey and Schofer, 2013). This neglects the crucial differences between the levels of engagement of different members (e.g., Painter and Paxton, 2014; Binder, 2021), the qualitatively different roles they play within associations (e.g., Kellerhals, 1974), and the different knowledge and skills they are endowed with prior to their membership (e.g., Miller, 2010; Passy and Monsch, 2020), which can have a significant impact on the form and magnitude of political participation effects they experience. Political participation is also often operationalized through one or two dimensions (e.g., Dini et al., 2014), which neglects the wide variety of forms of political engagement available to citizens today (van Deth, 2014; Theocharis and van Deth, 2018). At the same time, most of this literature is based on cross-sectional studies that correlate associational involvement with political participation after controlling for observable confounders. Since unobserved personality traits affect both sets of variables, and reverse causality is a serious problem because associational involvement and political participation mutually influence each other, cross-sectional studies are subject to severe endogeneity bias (e.g., van Ingen and Bekkers, 2015; Binder, 2021). In sum, the “average” picture that emerges from most of the existing research on the relationship between voluntary associations and political participation does not do justice to the great underlying heterogeneity and endogeneity of the relationship, which is stratified by type of association, level of involvement, pre-membership political capital, and forms of political participation.

This paper addresses all of these issues. It takes full advantage of the richness of information and the longitudinal nature of the Swiss Household Panel (SHP) (Voorpostel et al., 2024) by providing a systematic, nationally representative analysis of the extent to which different types of associational contexts may have heterogeneous effects on the political participation^1^ of individuals with different levels of education in Switzerland. To what extent does active involvement in associations have different political effects for individuals with different levels of education? Do these differences depend on the type of associational context and the form of political participation? Following the standard socioeconomic model (Verba et al., 1995), we consider education as a crucial stratification variable that affects both the propensity to engage in any form of collective action and political activity. We zoom in on how active involvement in voluntary associations can bridge the educational gap in different forms of political engagement. Leveraging the almost unique variety of variables on associational activities and level of involvement available in the SHP data, we consider a ternary conceptual model of civil society that distinguishes between expressive, instrumental, and advocacy associations (Cohen and Arato, 1994), and consider active involvement as a necessary condition for associations to have political effects. Again drawing on the richness of the SHP data, we conceptualize political participation as a multifaceted phenomenon that includes attitudinal forms of engagement, institutional political activity, community-oriented engagement, and protest and consumerist forms of participation (van Deth, 2014; Theocharis and van Deth, 2018). By focusing on the effect of the transition to associational activism on political participation, we exploit the panel structure of the SHP to control for all forms of observed and unobserved time-invariant heterogeneity.

Existing longitudinal studies of the effects of associational involvement on various political outcomes emphasize the importance of both selection and socialization effects (McFarland and Reuben, 2006; McAdam and Brandt, 2009; Hooghe and Quintelier, 2013; Quintelier, 2015; van Ingen and Bekkers, 2015; van Ingen and van der Meer, 2016; Wiertz, 2016; Binder, 2021; Hadziabdic, 2022). Selection refers to the fact that politically active individuals are more likely to join associations, while socialization refers to the way in which the experience of associational involvement increases political participation. When controlling for time-invariant heterogeneity, the socialization effects tend to be smaller than those found in cross-sectional studies. Nevertheless, even in panel data studies, the predominance of selection or causal effects varies considerably depending on the type of association, the characteristics of those involved, and the type of political outcome considered. Some studies show that involvement in youth associations fosters civic attitudes (Hooghe and Quintelier, 2013) and behavioral political engagement (McFarland and Reuben, 2006; Quintelier, 2015), while others point to selection effects as the main source of differences between activists and nonactivists (McAdam and Brandt, 2009). Other research focusing on various types of associations suggests that the link between political discussion, interest, efficacy, and trust and involvement in associations among the general population is mainly due to selection effects (van Ingen and Bekkers, 2015; van Ingen and van der Meer, 2016). However, active involvement in associations—as opposed to mere membership—is more likely to be associated with causal socialization effects on political participation (Binder, 2021; Hadziabdic, 2022). This paper builds on and extends this body of work by examining all these forms of heterogeneity at the same time and considering pre-membership political capital through education as a key stratification variable.

The remainder of the paper is structured as follows. After explaining why education is a crucial stratification variable that affects both associational involvement and political engagement, we elaborate on the reasons why associations can function as “schools of democracy” that, depending on the type of associational context and forms of political participation, can bridge or exacerbate the gap in political participation across educational levels. After describing the data and methodology, we present and discuss the empirical results. Finally, we highlight the main limitations of the paper and avenues for future research it opens.

## 2 Theoretical framework: bridging or exacerbating the political participation gap?

### 2.1 The standard socioeconomic model and the “schools of democracy” perspective

According to the standard socioeconomic model, a higher level of education provides individuals with knowledge, organizational skills, civic attitudes, and a network of like-minded peers that make them more likely to engage in society in general and the political sphere in particular (Verba et al., 1995; Warren, 2001). Education has been shown to be the strongest and most consistent sociodemographic predictor of all types of associational involvement (Kellerhals, 1974; Verba and Nie, 1987). At the same time, the same resources associated with education are also conducive to political engagement, not only conventional institutional participation but also new forms of political activity (Theocharis and van Deth, 2018).

Existing research, mostly based on cross-sectional data and/or case studies, finds that voluntary associations have strong positive effects on political attitudes in general and political participation in particular. Dating back to de Tocqueville (2016) writings on the crucial role of civil society associations in the vibrancy of American political life, associations are said to act as “schools of democracy,” instilling the political attitudes relevant to being active citizens in a democratic system in their members. We can distinguish four main mechanisms through which associations can influence political participation: knowledge, skills, civic attitudes, and social networks. Associations can increase factual knowledge about the political world by acting as information providers, whether through interaction with other members, the discourse of leaders, or the provision of newsletters (Mcclurg, 2003; Lamare, 2010; Kim and Margalit, 2017). Political knowledge promotes political engagement by reducing the costs of participation in terms of time and psychological strain, and by highlighting the rational benefits of political efficacy. Active involvement in the internal dynamics of associations can also improve the organizational skills needed in the political world (e.g., Quintelier, 2008; Baggetta, 2009). These skills also reinforce the sense that personal political engagement can make a difference (Verba et al., 1995). At a deeper level, active organizational engagement can influence civic attitudes by teaching members the importance of contributing to the common good, thus making political participation a normatively valued activity (Putnam, 2000; Lazer et al., 2010; Passy and Monsch, 2020). In addition to peer pressure, membership creates bonds that can be cultivated outside the association and increase the likelihood of being recruited for political action (Scheufele et al., 2004; Diani, 2007; Passy and Monsch, 2020). Active membership is a necessary condition for these mechanisms to be substantially important (Almond and Verba, 1963; Howard and Gilbert, 2008; Terriquez, 2011; Binder, 2021). The frequency of contact and the political knowledge of those interacting are particularly relevant (Lake and Huckfeldt, 1998; Eveland and Hively, 2009), especially for time-consuming political endeavors such as campaigning and protesting (Ayala, 2000).

While these mechanisms are often described as if they apply homogeneously to all associational contexts, other research points to a strong heterogeneity in both the antecedents and consequences of involvement in different types of associations (e.g., Stolle and Rochon, 1998; van der Meer and van Ingen, 2009; Häuberer, 2014). We conceptualize this heterogeneity by adopting the framework of civil society developed by Cohen and Arato (1994). Civil society is understood as a sphere at the intersection of the private, political, and public spheres. As a result, associations can be categorized into three main types: expressive, when their primary aim is to satisfy the psychological need for belonging of their members; instrumental, when they mainly exist to represent the rational interests of their members; or advocacy, when their raison d’être is to defend and promote public goods. All three types of associations can potentially influence political participation, but the different mechanisms we described are likely to be of different importance for each type. We therefore implicitly assume that the different dynamics that develop in the different types of associations are the moderating variable that links associational involvement and political participation.

Among expressive associations, despite their apolitical nature, sports clubs have been described as important socialization venues in youth that increase political engagement in adulthood (Donovan et al., 2004; Seippel, 2006). Face-to-face interactions and governance experiences in artistic and cultural associations have been associated with positive effects on political participation (Bowler et al., 2003; Dini et al., 2014) and civic attitudes (Baggetta, 2009). Instrumental associations that focus on narrow interests, such as those defending the rights of tenants or homeowners, can have negative effects on civic attitudes (McKenzie, 1996), while the broader interests represented by trade unions are associated with higher political participation (e.g., D’Art and Turner, 2007; Lamare, 2010; Kerrissey and Schofer, 2013). In recent decades, advocacy associations have been characterized by the rise of “checkbook membership” (Skocpol, 1999; Painter and Paxton, 2014), meaning that most members have a passive relationship with these organizations, paying dues to represent a cause they believe in but rarely actively participating in organizational activities. However, for the minority of members who do become involved, charitable groups have large positive effects on political engagement (Bowler et al., 2003). Because they function democratically, environmental organizations attract members who self-select into them because they value such practices, thereby reinforcing these individuals’ democratic engagement (Selle and Strømsnes, 1998).

In sum, while the standard socioeconomic model emphasizes that education is the most important predictor of any form of collective action, the “schools of democracy” framework predicts that active involvement in associational activities can bridge pre-membership differences between members from different social strata. However, existing research provides only scattered evidence on whether involvement in associations can reduce or exacerbate initial differences between members with different levels of cultural and political capital. In the next two subsections, we derive three general hypotheses about the circumstances under which associations can bridge or exacerbate the participation gap between individuals with different levels of education, and the extent to which these effects depend on the particular form of political participation.

### 2.2 Associational involvement: differential effects by education

Considering the heterogeneous effects of involvement in associations by education level, the most straightforward expectation is that individuals with lower qualifications experience the greatest increase in political participation. Compared to the more educated, the less educated are less likely to be knowledgeable about the political world, to have organizational skills, to see political participation as a social norm, and to be part of networks with individuals who are politically active (Verba et al., 1995; Warren, 2001). For this reason, they can benefit much more from their involvement in associations and have more scope to increase their political participation (Verba and Nie, 1987; Verba et al., 1995). Of the above mechanisms, the information channel has been described as the easiest way to trigger political interest and participation, as it only requires exposure to new information (e.g., Kerrissey and Schofer, 2013; Kim, 2014; Kim and Margalit, 2017).

*H1: The positive effects of associational involvement on political participation are more pronounced among the less educated.*

However, being exposed to political information does not always mean that the information is understood and welcomed (Zaller, 1991). If members of associations with low levels of education have a strong aversion to the political world, they may reject the opportunity to assimilate new information (Eveland, 2004). Because they feel less comfortable in an associational context due to the lack of personal resources, they may also be less willing to volunteer for skill-enhancing organizational tasks and to expand their network to include more educated members (Stoll, 2001; Li et al., 2005; Miller, 2010). On the other hand, highly educated individuals tend to self-select into associations that provide the most skills, and association leaders may rationally prefer to assign the most demanding tasks to highly educated individuals who already possess the necessary skills (Miller, 2010). If these tasks constitute the bulk of the activities that active members perform in a particular association, higher levels of education may even be a necessary condition for becoming an activist, leaving (most) low-educated members unable to become fully actively involved. In such a context, highly educated activists with already high levels of political participation would further increase their political engagement by finding a network of like-minded individuals who place a high value on political participation (Hooghe, 2003b). Thus, associational involvement may follow a Matthew effect, leading already advantaged individuals to make greater use of the opportunities offered by associations.

*H2: In contexts where high levels of knowledge and skills are required, associational involvement exacerbates the political participation gap between individuals with different levels of education.*

In trying to understand which of the three types of associations we focus on is most likely to bridge or exacerbate the gap in political participation across education levels, on the one hand, we may expect the learning effects for activists with low levels of education to be stronger in associations where political messages are explicit and frequent (Sobieraj and White, 2004). Because instrumental and advocacy associations have goals that are more clearly aligned with the political sphere than expressive associations, they are most likely to promote discussions and activities related to the political world. At the same time, instrumental and advocacy associations are also the contexts most likely to require and provide opportunities for skill enhancement (Miller, 2010; Lichterman and Eliasoph, 2014), increasing the likelihood that highly educated activists are most likely to capitalize on these opportunities. By contrast, despite their apolitical nature, the higher membership heterogeneity and lower skill requirements of the internal dynamics that characterize expressive associations, such as sports and cultural associations, may give them an advantage in bridging differences in political participation (Bowler et al., 2003; Baggetta, 2009; Dini et al., 2014). In sum, the different traits of the three types of associations lead to contrasting expectations regarding their ability to reduce or increase differences in political participation among active members with different levels of education. When discussing the empirical patterns presented below, we implicitly assume that associational types can serve as proxies for different internal dynamics. However, as we detail in the concluding section, this is a speculative exercise that should be supplemented with direct measures of internal dynamics within single associations as experienced by individual members. The results presented below suggest the existence of different associational subtypes, particularly among expressive associations that individuals with different levels of education are most likely to join.

### 2.3 Varieties of political participation: a ladder of political participation?

Besides being interested in the heterogeneous effects of associational involvement by education and by type of association, we also assume the existence of heterogeneous effects depending on the form of political participation considered as outcome. The fact that political participation can take many forms other than voting has already been emphasized decades ago in classical works (e.g., Almond and Verba, 1963). In this paper, we focus on and extend the framework developed by van Deth (2014), which has been refined and empirically measured in Theocharis and van Deth (2018). Van Deth’s (2014) crucial observation is that non-political acts can have political goals. He provides a broader definition of political activities as all those behaviors that individuals engage in voluntarily, in their role as citizens, with the intention of influencing the government/state/political sphere and/or collective/community issues. Although van Deth’s model does not consider attitudinal dimensions as a form of political participation, it makes sense for our purposes to include them as well. Indeed, knowledge and civic attitudes are among the crucial mechanisms we mentioned above through which associational involvement affects political participation. Attitudes have also been shown to be strong behavioral precursors (Ajzen, 1991). By extending van Deth’s model to include attitudinal dimensions, we consider six forms of political engagement. First, attitudinal dimensions such as interest in politics or feelings of political efficacy are crucial antecedents of behavioral political participation. Second, conventional institutional political participation includes behaviors such as voting in elections and referendums, party membership, and contacting politicians. Unconventional political participation, such as petitions, protests, strikes, and demonstrations, is a third form that aims to circumvent traditional institutional channels. Fourth, civic engagement, such as participation in community activities and volunteering, affects the political world through the channel of the local community. Fifth, individualized and personalized forms of political expression, such as boycotts and buycotts, enable citizens to take political action through their choices as consumers. Finally, digitally networked participation through social media and online activities is a form of participation that has recently become prominent. Based on personalized media consumption and networking with imagined rather than physical communities, this type of participation has the potential to disrupt the way citizens approach the political world (Vromen, 2017).

In their empirical examination of this taxonomy, Theocharis and van Deth (2018) show that the different forms of political participation are correlated with each other. This correlation means that new, more complex forms of participation tend to be an extension of traditional political activities. Most citizens take part in institutional electoral participation. A decreasing proportion of these citizens also participate in unconventional forms of participation, followed by consumer-related participation and civic engagement. In other words, there is a “ladder of political participation,” which means that certain forms of participation are more demanding than others. Because some forms of participation are more exclusive and complex than others, we assume that higher individual resources are required to participate in them. Given pre-existing differences in educational attainment and the fact that responsibilities within associations are unevenly distributed between low- and high-skilled individuals (Miller, 2010), we expect that highly educated active members of associations are more likely to be involved in internal associational dynamics that are sophisticated enough to influence more complex forms of political participation.

*H3: The more complex the form of political participation, the less able associations are to bridge the participation gap between individuals with low and high levels of education.*

Interestingly, Theocharis and van Deth (2018) also show that the antecedents of the different forms of participation, in particular the civic value of good citizenship associated with political participation, are the same. This corroborates the pertinence of our decision to also consider attitudinal dimensions as political outcomes.

## 3 Data and methodology: addressing heterogeneity and endogeneity

### 3.1 Data: the Swiss household panel

In order to examine the heterogeneous effects of associational involvement by educational level on different forms of political participation, we make use of the wealth of information available in the Swiss Household Panel. Due to the high statistical power required by our estimation strategy (see next subsection), we restrict our focus to independent and dependent variables that are available in most survey waves, thus allowing us to cover the period between 1999 and 2020. As key independent variables, we focus on six types of associations. Following the framework of Cohen and Arato (1994) described above, we consider two expressive (sports clubs, cultural associations), two instrumental (local interest groups,^2^ trade unions^3^), and two advocacy (environmental organizations, charitable associations) associations. While the SHP data provide information on passive and active membership in these associations, the mechanisms we postulated above to explain the differential effects of associations on political participation by education require active involvement in associations. We therefore focus on active membership in these associations as the explanatory variable.

As dependent variables, we focus on the taxonomy of political participation described above (van Deth, 2014; Theocharis and van Deth, 2018). As attitudinal dimensions of political engagement, we consider interest in politics and the feeling of political influence. As electoral/institutional forms of participation, we include participation in federal polls and membership in political parties.^4^ As fifth dependent variable, being active in voluntary work operationalizes a community-oriented form of political participation highly congruent with associational dynamics (Binder, 2021).^5^ The propensity to participate in future demonstrations and the likelihood of engaging in future boycotts are associated with protest and consumerist forms of political engagement, respectively.^6^

The link between these independent and dependent variables is interacted with a ternary indicator representing the highest level of education attained: primary education or less; secondary education; higher vocational or academic tertiary education. Using cross-sectional weights, these categories cover 24, 55, and 21% of the Swiss population, respectively. The dominance of secondary education reflects Switzerland’s specificity as a coordinated market economy in which firm-based vocational training plays an important role (Busemeyer and Trampusch, 2011). The original survey questions and the operationalization of all independent and dependent variables, as well as the key educational stratification variable, can be found in Supplementary Table A1.

In addition to these variables, our modeling strategy includes a set of standard time-varying control variables (see next subsection): age (as a linear and quadratic term), nationality, region of residence, couple status, number of children in the household, working status, and time fixed effects. The latter are intended to capture any societal period effects that may affect the relationship between associational involvement and political participation. Descriptive statistics for all variables are presented in Supplementary Table A2.

### 3.2 Methodology: within-individual change

To examine the heterogeneous relationship between associational involvement and political participation by educational level, we focus on the following specification:


(1) $$\begin{array}{l} P_{\mathrm{it}}=\alpha +\beta A_{\mathrm{it}}+E\prime_{\mathrm{it}}\delta +(A_{\mathrm{it}}xE_{\mathrm{it}})\prime \zeta +C_{\mathrm{it}}^{\prime }\eta +\nu_{i}\\ +\omega_{\mathrm{it}},\text{for}\;i=1,2,\ldots ,N\;\text{and}\;t=1,2,\ldots ,T \end{array}$$

where i and t are indices associated with individuals and time periods, respectively; P_it_ is the political participation outcome of interest; *α* is the intercept common to all individuals and time periods; A_it_ is a binary indicator coded 0 if an individual is not a member of the association of interest and 1 if they are an active member, and *β* its associated estimate; E_it_ is a vector representing the categorical variable associated with the three modalities of educational attainment, along with their estimates *δ*; A_it_xE_it_ is the interaction term between active associational membership and education level, along with their estimates *ζ*; C_it_ is the vector of the control variables described in the previous subsection and *η* their associated estimates; ν_i_ is a time-invariant error term that varies only between individuals; ω_it_ is an error term that varies over time as well as between individuals.

We estimate the model described in equation (1) using the fixed effects estimator, i.e., applying Ordinary Least Squares (OLS) to time-demeaned data.^7^ This means that we focus on how within-individual changes in associational involvement by education are associated to changes in political participation after controlling for the effects of the control variables. This strategy also allows us to control for the individual time-invariant fixed effects ν_i_, which in particular represent unobserved personality traits that are likely to simultaneously affect associational involvement, educational attainment, and political participation. The latter is an important source of endogeneity that cannot be controlled for in the existing, mostly cross-sectional research. Nevertheless, we are cautious about interpreting the empirical patterns described below in causal terms. This is because we are still exposed to time-varying endogeneity. Since both associational involvement and political participation are related to engagement in the public sphere, time-varying reverse causality is a serious source of bias, especially in the case of politicized associations (e.g., van Ingen and Bekkers, 2015; Binder, 2021).

Since the paper focuses on the heterogeneous effects of associational involvement by education level, and since our estimation strategy only considers within-individual variation, it is crucial to keep educational attainment constant. For individuals who change the highest level of achieved education during their participation in the SHP, we ensure time-invariance by only considering the highest level reported across all participation waves and treating all responses reporting a lower level as missing data. This ensures that the estimate of the interaction term is influenced solely by changes in associational involvement, rather than by changes in the educational qualifications of young respondents. This also avoids the complex problems associated with interpreting the interaction between two time-varying variables in a fixed effects model (Giesselmann and Schmidt-Catran, 2022). Similarly, as only 0.08% of respondents experienced a change in gender during their participation in the SHP, we treated gender as time-invariant by considering only the final gender reported for these individuals and removing observations with the originally reported gender. As at least 1% of respondents experienced at least one within-individual change in the modalities of the other control variables, we considered these as sufficiently time-varying and did not apply any restrictions.

Since we are interested in the effects of the transition from non-involvement to active associational involvement, and to avoid having to assume that the transition from nonmember to active member has the same opposite effect as the transition from active member to nonmember, we limit our focus to the transitions from nonmember to active associational involvement and exclude those that represent the opposite transition. More specifically, to limit our focus to transitions from nonmember to member, we set all observations after an individual leaves an association as missing. For example, if an individual is a nonmember between 1999 and 2002, a member between 2003 and 2007, and a nonmember between 2008 and 2022, all observations from 2008 onwards are set as missing, as existing research shows that the effects of associational involvement may last years after leaving (Hooghe, 2003a). This is similar to the principle behind estimating asymmetric fixed effects (Allison, 2019), but we consider our approach to be more suitable for the purposes of this paper, since it is simpler and does not require any assumptions, unlike Generalized Least Squares (GLS) estimation in asymmetric fixed effects.

## 4 Empirical findings: selection and treatment effects

### 4.1 Descriptive analyses: membership gap not translating into activism gap

**Figure 1:**
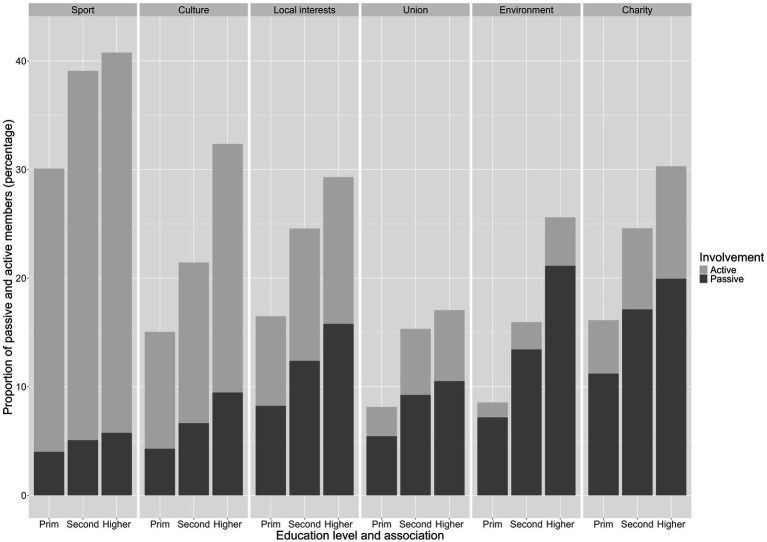
Propensity to join different types of associations by level of education.

Before addressing the question of whether associational involvement leads to changes in political participation, we examine the differences in associational membership and political engagement by educational attainment. Because these are descriptive analyses, all analyses in this subsection are weighted with cross-sectional weights. In Figure 1, we look at the propensity to join associations. The barplot shows the share of passive and active members for each of the six types of associations, broken down by education level (see Supplementary Table A3 for the exact underlying percentages). The first aspect to note is that, as expected, there are significant differences between the types of associations, both in terms of propensity to join and active involvement. As the most democratic form of associational involvement (Seippel, 2006), at least a third of individuals in all three education groups are members of a sports club, most of them as active members. Cultural, local interest, and charitable associations follow in terms of membership and the share of active members. Trade unions are the association with the lowest proportion of members across all education levels (between 8 and 17%), with about half of them being active members. Environmental organizations have slightly higher membership rates (between 9 and 26%, depending on the level of education), but are also the type of association with the highest proportion of passive members (4 out of 5). Indeed, environmental organizations have been described as the archetypal case of checkbook membership (Skocpol, 1999; Painter and Paxton, 2014). Run by professional recruiters in distant headquarters, they mostly consist of members who soothe their conscience primarily by contributing financially to a cause in which they believe. The other obvious pattern is the presence of a consistent difference between passive and active membership according to level of education: the higher the level of education, the more likely one is to be a passive and active member, regardless of the type of association. For example, 30% (4% passive; 26% active) of those with primary education are members of a sports club, while the figure is 41% (6% passive; 35% active) for those with tertiary education. The difference is even more pronounced among trade union members: 9% (5% passive; 3% active) of those with a low level of education are members, compared to 17% (11% passive; 7% active) of those with a high level of education.

**Figure 2:**
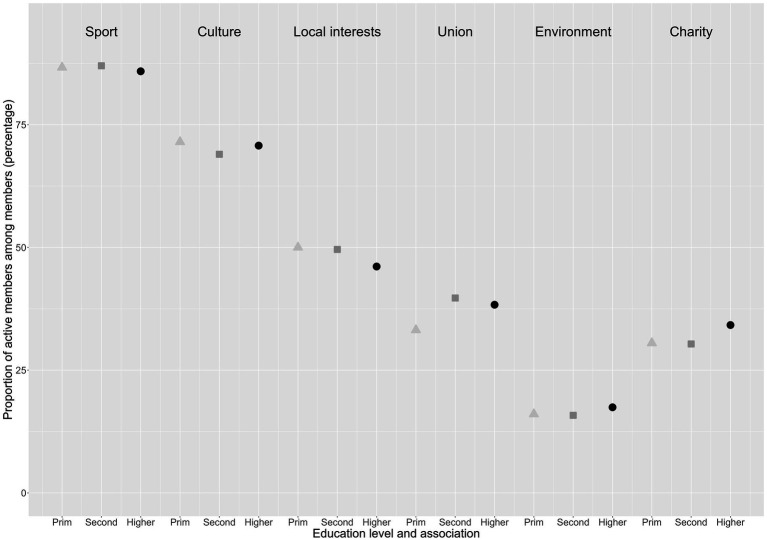
Propensity to be actively involved in different types of associations by level of education.

While there is a clear differential in the likelihood of joining an association depending on the level of education, in Figure 2 we look at the propensity to be actively involved in associations. We zoom in on whether this difference also affects the propensity to be active in associations (see Supplementary Table A4 for the exact underlying percentages). In other words, we calculate the proportion of active members among the members (passive or active) of each type of association. The picture changes radically and the differences between educational levels become very small. In the two expressive associations, members with a low level of education are a few percentage points more likely to be active than the other two categories (e.g., sports clubs: 87% active members with primary education; 86% active members with higher education). In local interest groups and unions, members with secondary education are slightly more likely to be active members than those with tertiary education. It is only in the two advocacy associations that members with a higher education are slightly more likely to be active members, but this advantage is much smaller (1–3%) than the advantage in terms of overall willingness to join that can be seen in Figure 1. In other words, Figure 1 confirms that a higher level of education confers a clear advantage in the likelihood of joining an association of any type. However, when we control for this baseline difference and focus only on those who actually joined in Figure 2, the advantage of the highly educated in terms of propensity to become actively involved tends to disappear and in some cases is even reversed.

**Figure 3:**
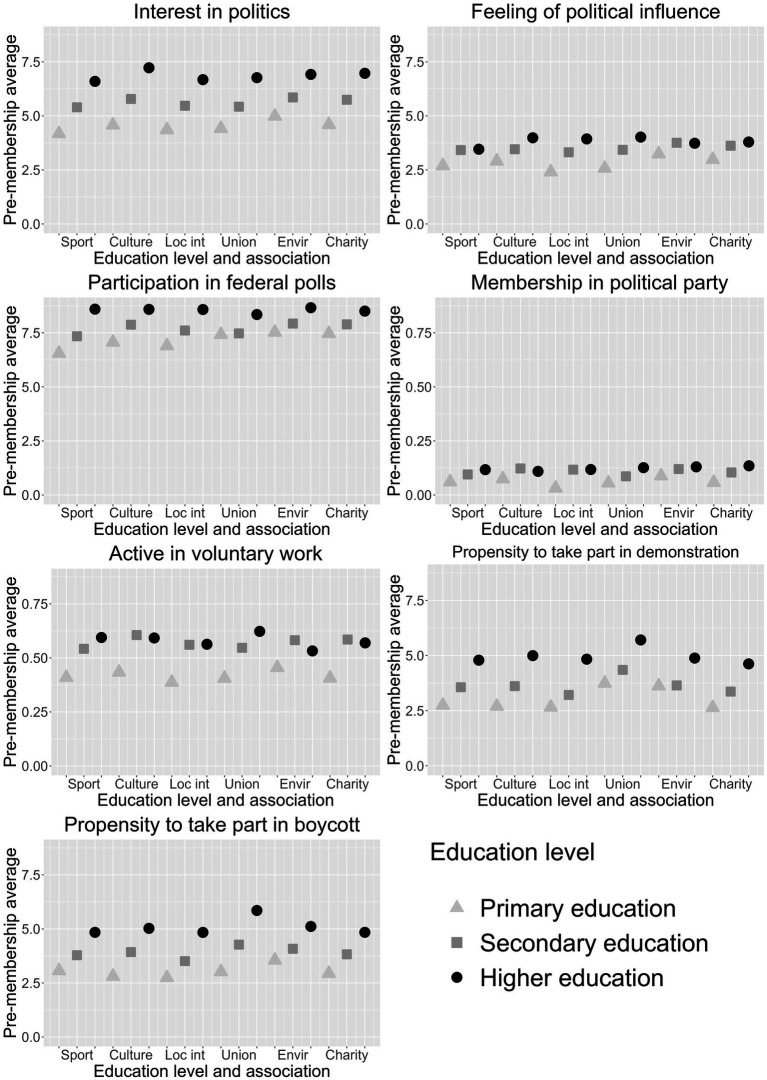
Pre-membership average in different forms of political participation by associational type and level of education.

In the next subsection, we test whether this same level of active associational involvement by education level is associated with similar effects on different forms of political participation. Before doing so, we examine in Figure 3 the level of political engagement of individuals 1 year prior to joining different associations by educational level (see Supplementary Table A5 for the exact underlying numeric values). In other words, we focus on the average level prior to joining in the dimensions of political participation of interest, by type of association and level of education. First, we see clear differences in the average value of the different forms of political participation, regardless of the associational context or level of education. Participation in federal polls has the highest averages, with values between 7 and 9, which is at least partly related to the fact that institutional political participation is most affected by social desirability bias in survey responses (Goldberg and Sciarini, 2019). The other form of institutional participation, political party membership, shows the lowest values, meaning that in no case is the membership rate higher than 13%. Interest in politics prior to membership varies between 4 and 7. These averages are higher than the 2–4 values visible for the feeling of political influence. The averages for the propensity to participate in demonstrations and boycotts are between 3 and 6, while the propensity to be active in voluntary work before joining an association concerns between 39 and 62% of individuals, depending on the type of association and the level of education. Second, with a few exceptions (e.g., prospective members of some associations with secondary education are more likely to volunteer), we find a clear educational gradient: individuals with higher levels of education are more engaged in almost all forms of political participation regardless of the type of association. Third, it is also clear that these pre-membership averages are mainly determined by the form of political participation and the level of education, and not by the type of association, since most associations have similar averages for the same outcome variable by education level. To sum up, there are clear differences in the average level before active involvement in associations in the different forms of political participation, which almost always involve higher values for highly educated prospective members. This makes them more politically active than the other education groups even before they join, which may limit the scope for further increases in political engagement, but it may also make them more open to further political activity.

### 4.2 Fixed-effects estimates: narrowing or widening the political participation gap?

**Figure 4:**
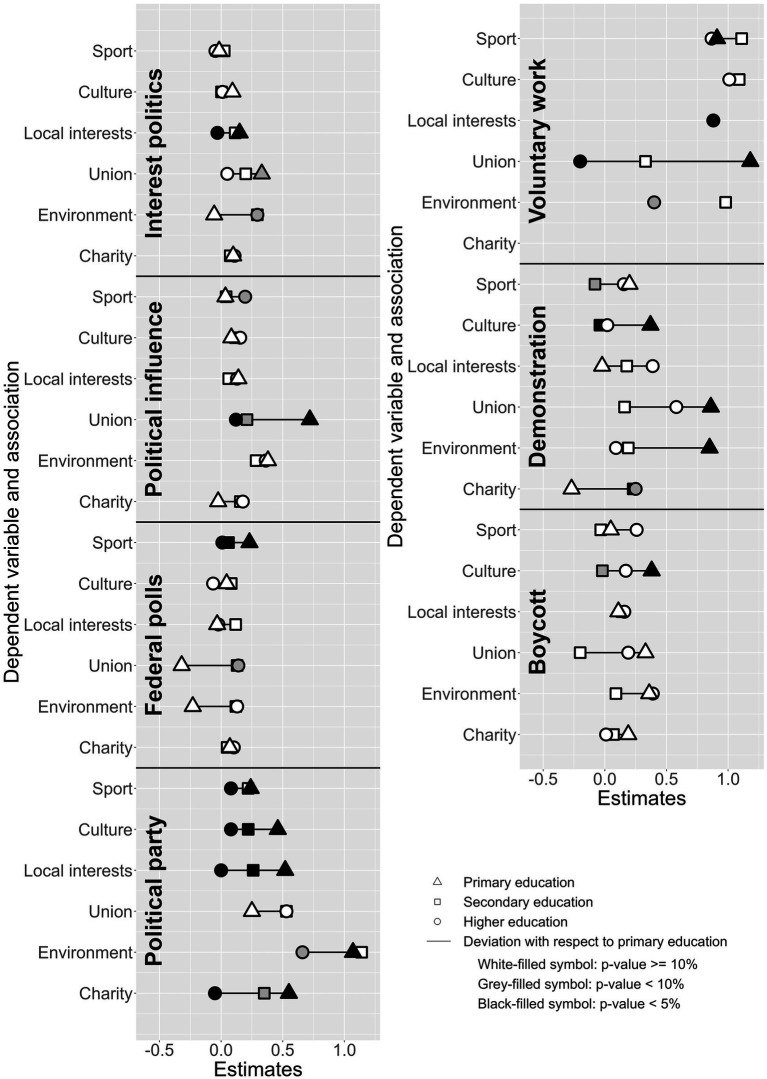
Fixed-effects estimates of the impact of being active in different types of associations on different forms of political participation interacted with education level.

In the specification in equation (1), the reference category is represented by nonmembers of the association of interest with primary education. We are mainly interested in the estimate for active members with primary education (which captures the effect of active associational involvement among individuals with primary education) and the deviation from this effect among individuals with secondary and higher education. We plot these three estimates in Figure 4 for each pair of independent (six types of associations) and dependent (seven forms of political participation) variables we focus on, and indicate their statistical significance by the color of the symbols representing them. Because five of the dependent variables are measured on a 0–10 scale, the two binary (0, 1) outcomes (political party membership, volunteering) were graphically rescaled to the same 0–10 scale and the x-axis boundaries standardized to [−0.5, 1.2] to make the magnitude of the estimates visually comparable. The full models with estimates for all remaining control variables, as well as the standard errors, exact *p*-values, and 95% confidence intervals of all estimates, are available in Supplementary Tables B1–7. Before commenting on the empirical patterns, we emphasize that despite the quality and large number of observations provided by the SHP data, we are likely to face problems of statistical power. We focus on within-individual variation in active associational involvement interacted with three levels of education. Some associations, especially environmental organizations, have a limited number of active members. At the same time, the three levels of education were defined on the basis of substantive considerations and vary in size, so that we have more statistical power for estimates related to secondary education than for those related to higher education.

Considering interest in politics as the dependent variable, we find that in two associations (union: 0.33, *p* < 0.10, CI^8^ [−0.029, 0.69]; local interests: 0.15, *p* < 0.05, CI [0.0074, 0.29]), there is a significant positive effect among activists with primary education. In local interest groups (−0.18, *p* < 0.05, CI [−0.36, −0.012]), these effects are significantly larger than for activists with higher education. Additionally, we observe a significantly greater increase in political interest among individuals with secondary (0.35, *p* < 0.10, CI [−0.0011, 0.71]) and higher (0.35, *p* < 0.10, CI [−0.039, 0.74]) education in environmental organizations than among activists with primary education. Regarding the second dimension of attitudinal political engagement, we find that the feeling of political influence is significantly positively affected for trade union activists with low levels of education (0.72, *p* < 0.01, CI [0.21, 1.22]), an effect significantly higher than those found among activists with secondary (−0.51, *p* < 0.10, CI [−1.05, 0.019]) and tertiary (−0.60, *p* < 0.05, CI [−1.16, −0.036]) education. By contrast, individuals with higher education experience a significantly greater increase in political influence when they are active in sports clubs than those with primary education (0.16, *p* < 0.10, CI [−0.028, 0.34]).

Turning to the institutional forms of political engagement, we find a positive effect on participation in federal polls among active members with a low level of education in sports clubs (0.23, *p* < 0.01, CI [0.089, 0.36]), which is significantly less pronounced for members with secondary (−0.17, *p* < 0.05, CI [−0.32, −0.025]) and tertiary (−0.22, *p* < 0.05, CI [−0.38, −0.048]) education. The opposite pattern is found for trade unions, with a significantly more positive effect for active members with secondary (0.45, *p* < 0.10, CI [−0.077, 0.98]) and higher (0.46, *p* < 0.10, CI [−0.088, 1.01]) education. When we focus on the probability of joining a political party, we find that active members with primary education experience a significant increase in all types of associations (environment: 0.11, *p* < 0.001, CI [0.072, 0.14]; charity: 0.055, *p* < 0.001, CI [0.037, 0.074]; local interests: 0.052, *p* < 0.001, CI [0.035, 0.069]; culture: 0.046, *p* < 0.001, CI [0.032, 0.061]; sports: 0.024, *p* < 0.001, CI [0.012, 0.036]) except trade unions. In three of these associations, these effects are significantly higher than for active members with secondary education (local interests: −0.026, *p* < 0.01, CI [−0.044, −0.0072]; culture: −0.024, *p* < 0.01, CI [−0.041, −0.0076]; charity: −0.020, *p* < 0.10, CI [−0.041, 0.000066]), while in five of them they are significantly more pronounced than for activists with higher education (charity: −0.060, *p* < 0.001, CI [−0.083, −0.036]; local interests: −0.052, *p* < 0.001, CI [−0.073,-0.032]; environment: −0.041, *p* < 0.10, CI [−0.083, 0.0016]; culture: −0.038, *p* < 0.001, CI [−0.056,-0.019]; sports: −0.16, *p* < 0.05, CI [−0.032, −0.00052]).

All associations increase the likelihood of volunteering among active members with low education (charity: 0.27, *p* < 0.001, CI [0.23, 0.31]; local interests: 0.19, *p* < 0.001, CI [0.15, 0.23]; culture: 0.13, *p* < 0.001, CI [0.097, 0.17]; environment: 0.13, *p* < 0.01, CI [0.041, 0.22]; union: 0.12, *p* < 0.001, CI [0.0075, 0.23]; sports: 0.091, *p* < 0.001, CI [0.064, 0.12]). For the two associations with the largest effects (which lie outside the limits of the x-axis), these effects are significantly more pronounced than for individuals with secondary education (charity: −0.076, *p* < 0.01, CI [−0.12, −0.029]; local interests: −0.064, *p* < 0.01, CI [−0.11, −0.022]), while for four associations they are greater than for individuals with tertiary education (union: −0.14, *p* < 0.05, CI [−0.26, −0.017]; local interests: −0.099, *p* < 0.001, CI [−0.15, −0.052]; environment: −0.091, *p* < 0.10, CI [−0.20, 0.014]; charity: −0.080, *p* < 0.01, CI [−0.13, −0.027]).

Looking at the propensity to take part in future demonstrations as a form of protest behavior, it increases significantly for activists with low levels of education in unions (0.86, *p* < 0.05, CI [0.079, 1.63]), environmental organizations (0.85, *p* < 0.05, CI [0.091, 1.62]), and cultural groups (0.37, *p* < 0.05, CI [0.058, 0.68]). For cultural groups, this positive effect is significantly more pronounced than for those with secondary education (−0.41, *p* < 0.05, CI [−0.76, −0.064]), a pattern that is also observed for sports clubs (−0.28, *p* < 0.10, CI [−0.58, 0.012]). In charitable associations, on the other hand, those with secondary (0.50, *p* < 0.05, CI [0.044, 0.96]) and higher (0.52, *p* < 0.10, CI [−0.048, 1.09]) education are significantly more likely to increase their likelihood of taking part in demonstrations than those with the lowest level of education. Finally, the probability of participating in future boycotts is significantly affected only for activists with low qualifications in cultural groups (0.38, *p* < 0.05, CI [0.020, 0.73]), an effect significantly more pronounced than among individuals with secondary education (−0.40, *p* < 0.10, CI [−0.79, 0.00058]).

## 5 Discussion: when and how individual profiles, associational contexts, and political outcomes match

With the aim to test whether active involvement in associations is related to educational attainment, we showed that higher levels of education are associated with a higher likelihood of joining all types of associations. This is consistent with the idea that education provides individuals with knowledge, skills, attitudes, and social networks that match what is expected in associational contexts (Verba et al., 1995), making it easier to join. Although these advantages of higher levels of education play an important role as selection mechanisms in associations, we demonstrated that they do not significantly affect the level of involvement in all types of associations. In other words, education is a powerful gateway factor that determines the likelihood of becoming a member of an association, but once an individual has joined, their level of involvement is largely independent of their educational attainment. In some cases, especially in expressive groups, members with lower levels of education are even slightly more active than members with higher levels of education, who have a marginally higher propensity to be actively involved only in advocacy organizations.

To find out whether education is also correlated with political participation, we examined the pre-membership averages across associational types in different forms of attitudinal, institutional, community-oriented, protest, and consumerist political engagement. As expected, these forms of political participation follow a ladder (Theocharis and van Deth, 2018), showing that political engagement in the form of voting in federal polls is the most widespread, followed by interest in politics and volunteering. Protest behavior in the form of demonstrations and boycotts is at a lower level, as is the sense of political influence. Political party membership remains a niche form of political participation, involving less than 10% of the population. These levels of political participation are also accompanied by a gradient of educational differences. For each of these forms of political engagement, a higher level of education is consistently associated with a higher level of engagement, and this is true of the pre-membership averages in all types of associations.

This leaves more room for growth in political participation for those with a low level of education. Consistent with this pattern, our first theoretical expectation implies that activists with the lowest levels of education can benefit more from associational involvement in terms of knowledge, skills, attitudes, and networking opportunities and should therefore experience stronger positive effects on political participation than active members with higher levels of education. The overall pattern of results obtained using fixed effects models confirms this expectation. Of the 24 significantly estimated associations between associational involvement and political participation, activists with a primary education or less show stronger positive effects than those with higher qualifications in 18 cases. This difference is strongest and most consistent across almost all types of associations when it comes to the likelihood of joining a political party, and even more so when it comes to volunteering. Since these forms of political participation are the ones most likely to be congruent with associational activities, we can assume that the knowledge, skills, and social ties that low-educated activists develop in the associational context are easily transferable to what is required to join parties and perform voluntary work. In the case of the link between volunteering and some associations, especially those of a charitable nature, the positive effect is so strong that it is likely to be related to reverse causality and/or to an overlap between activities in the association and volunteering. While the relationship between associational involvement and volunteering is, to some extent, tautological (Binder, 2021), the consistent associations we detect are useful in verifying that the self-evaluated active involvement measured through SHP data does indeed imply increasing direct participation in associational dynamics for a significant proportion of respondents. Taken together, these findings disappoint to some extent de Tocqueville’s and Putnam’s idealistic views of associations as “schools of democracy” (Putnam, 2000; de Tocqueville, 2016). While they describe associations as broadening the horizons of activists and fostering civic and democratic attitudes and behaviors, the strongest and most consistent effects of associational involvement manifest in niche, instrumental political participation, as is the case with most political parties, or in voluntary work activities that may be motivated by instrumental factors and potentially coincide with associational involvement.

Interestingly, in addition to an increased likelihood of volunteering, being active in trade unions is the type of associational involvement that leads to a greater willingness to become attitudinally involved in the political world, with an increase in interest in politics and the feeling of political influence, and to participate in demonstrations among low-educated members. In contrast, institutional political participation, such as voting in federal elections or joining a political party, remains unaffected. This could be related to the more precarious working conditions that low-educated workers are generally exposed to (Kalleberg, 2009), making them more likely to experience the union environment as an opportunity to protest outside institutional channels. A similar effect, albeit of smaller magnitude, can also be observed for cultural and environmental associations. Becoming an active member of a local interest group also triggers greater interest in politics among activists with a low level of education. Despite its apolitical nature, activity in sports clubs leads to a small but significant increase in the willingness to participate in federal polls, especially among members with a low level of education.

The second general expectation we identified concerns the presence of more pronounced effects of associational involvement for highly educated members when the associational context requires knowledge and skills mainly possessed by individuals with higher qualifications. Advocacy organizations such as environmental and charitable associations best fit this type of associational context. Highly educated activists in environmental organizations are more likely to become interested in politics as a result of their associational involvement than those with lower qualifications. The same is true of charitable associations with regard to the propensity to take part in demonstrations. Six additional estimates associated with these two types of associations point in the same direction, albeit not significantly. While the existing literature highlights the increasing passive checkbook membership of environmental organizations, these analyses, which focus on the small proportion of active members, emphasize that environmental and charitable activism are niche activities, often in the hands of professional campaigners who must be adept at running national headquarters and recruiting new (checkbook) members (Fisher and McInerney, 2012). It is therefore not surprising that most of the positive effects are concentrated among the highly educated, who are most likely to self-select into and to be entrusted with these activities (Miller, 2010). In a similar vein, sports clubs increase the feeling of political influence only among highly educated active members and the same applies to the positive effect of trade union activism on the participation in federal polls. We can interpret this finding as being related to the presence of sports clubs and trade unions that are almost exclusively joined by highly educated activists. It is in these associations with highly skilled members that some politically charged interactions can take place between individuals with high political capital who have the civic attitudes necessary to value political participation even in apolitical contexts (Hooghe, 2003b).

The third general expectation we have formulated relates to the ladder of political participation. We expected that the forms of political engagement that are least likely to occur in the general population are also those that are most difficult to influence through associational involvement and should follow a gradient by education level. Our results lead us to qualify this expectation in two ways. First, rather than ranking forms of political participation, it makes more sense to distinguish qualitatively between forms of political participation that are more or less congruent with the knowledge and skills developed in associational activities. Although party membership is the least common form of political participation, it is the outcome variable with the most consistent and strongest effects after volunteering, because they both have obvious similarities to associational structures and internal dynamics, and also likely overlap in recruitment networks. On the other hand, feeling politically influential, and especially the willingness to participate in boycotts, are the least influenced by involvement in associations across all educational groups. These are the dimensions that seem to be most resistant to change because they are high on the ladder of political participation. In addition, the activities taking place in associations are probably not congruent enough with what is necessary to influence these forms of political participation. In particular, associational experiences are forms of collective action, while boycotts are a highly individualized form of political participation (Neilson, 2010). Second, we found that the pre-membership level in a particular form of political participation also limits the scope for additional increases that an individual can experience. If an individual’s level of activity is close to or at the measured maximum threshold, there is not much to be gained after joining an association. This pattern is most evident for participation in federal polls, which, while showing some significant positive effects, is severely limited in scope by the fact that most prospective activists have a very high level on this dimension immediately prior to joining.

Finally, of the forty-two relationships we considered (active involvement in six types of associations across seven forms of political participation), 18 show no significant estimates, either in the reference category of activists with low levels of education or in the deviation of the effects in the other two education groups. On the one hand, there are substantive reasons for this, since associational involvement only has a significant effect on political participation if several conditions are met: the type of individual who is involved in an association needs to have sufficient scope to increase their political participation and the ability and willingness to do so, the type of associational context must provide experiences that can be used in the political sphere, and the form of political participation has to be congruent enough with associational dynamics. On the other hand, because we focus only on within-individual changes in activists stratified by educational groups, the statistical power required to achieve statistical significance is quite high, allowing us to achieve significance mainly for relatively large and consistent effects.

## 6 Concluding remarks: delving deeper into (causal) mechanisms and emerging forms of political participation

While our longitudinal approach provides stronger leverage in controlling for unobservable, time-invariant heterogeneity, we cautioned the reader multiple times that the effects we presented were, at least in part, related to self-selection rather than being causal. The predominance of causal over selection effects is likely to vary considerably according to the form of political participation considered. Volunteering is probably the most endogenous outcome we considered, and therefore shows the strongest link with associational involvement. Nevertheless, as existing research underlines (e.g., van Ingen and Bekkers, 2015; Binder, 2021), panel data remains the best option for estimating the causal effects of associational involvement on political attitudes as closely as possible. Indeed, alternative causal inference approaches are difficult to design, as valid instrumental variables are hard to find, and randomized or natural experiments based on observational data with real-world implications are hard to conceive (Meier and Stutzer, 2008).

Despite the richness of the SHP data, future research could go beyond the information on which we have relied in several respects. First, throughout this paper we have implicitly assumed that different types of associations can be used as proxies for different associational dynamics, which in turn explain why we observe certain effects on specific forms of political participation. In other words, we used the type of association to capture the mediating role of associational experiences in influencing different forms of political engagement. While this was the best we could do with the data at our disposal, existing research shows that heterogeneity in associational dynamics within the same associational types captured in survey data can be substantial and may even be greater than the heterogeneity between types we focused on (Baggetta and Madsen, 2019). A better approach would be to measure the actual type of activities and the time each activist spends on them, as has sometimes been done in dedicated surveys (Wollebæk and Selle, 2002). This information would be indispensable for identifying the real reasons why individuals with different levels of education experience different political effects in different associational contexts. For example, we have argued that the peculiar empirical pattern in environmental and charitable organizations, where highly educated members tend to experience greater increases in political participation, even in dimensions where they are *a priori* better off than other social classes, can be attributed to the more complex organizational dynamics in these associations, which are better suited to incorporate the skills associated with higher education. It would be better to test these mechanisms empirically rather than assume them. In this context, it would also be important to have more detailed information about individuals’ actual knowledge and cognitive and organizational skills, the extent to which they correlate with education, and how they can be influenced by associational experiences. Although we have studied different forms of political participation, their diversity is increasing, with digitally networked political engagement in particular gaining in importance (Theocharis and van Deth, 2018). Although some SHP waves provide information on how much time is spent online, we could not find any suitable variable with which to operationalize digital political participation. In light of the growing significance of digital citizenship as a vital form of political engagement that complements and supplants other modes of participation (Vromen, 2017), this is a significant limitation of our paper that must be addressed by future research.

One could also wonder to what extent the external validity of the empirical findings we described extends beyond the Swiss case. On the one hand, Switzerland is a country that has been shown to be characterized by a comparatively high level of associational involvement (van Deth and Maloney, 2014) and with particularly developed democratic institutions, in particular the presence of a channel of direct democracy (Vatter, 2020). Repeating the same analyses in countries with lower levels of social capital and political engagement would be an interesting avenue for future research, as the gap in political participation between individuals with different levels of education may be wider than the one described in this paper. On the other hand, Switzerland is a coordinated market economy in which the share of university graduates is lower than in most advanced countries (Busemeyer and Trampusch, 2011). This leads to a larger number of individuals with secondary education and makes tertiary education comparatively more “exclusive” than in other national contexts. This “issue” can be overcome by focusing on stratification variables other than education. We focused on educational attainment as the main source of heterogeneous effects because it is empirically parsimonious and directly related to the knowledge and skills needed for political participation. It might be interesting to go beyond the three categories examined in this paper or to focus on parental education as well, which is available in the SHP data. Apart from education, the multidimensionality of today’s political space might also be better captured by other indicators of social stratification, such as objective social class (Oesch, 2006) or indicators of subjective class affiliation, which are better suited to capture the interplay between cognitive (cultural) and material (economic) considerations that influence not only the level but also, and especially, the form of political participation (Oesch and Rennwald, 2018).

Finally, in this paper we focused on the average effects of a specific type of associational involvement trajectory by examining individuals who transitioned from nonmember to active member status. However, existing longitudinal research shows that pre-membership history, changing levels of involvement during the membership career, and duration of associational experience significantly impact the political effects of associational involvement (McAdam, 1990; Hadziabdic and Baccaro, 2020). Future research could use SHP data to identify the types of associational careers most likely to positively impact political participation.

## Data Availability

The data analyzed in this study is subject to the following licenses/restrictions: The data that support the findings of this study are based on the Swiss Household Panel (SHP). Researchers can have access to the data after signing an individual user contract with the Swiss Centre of Expertise in the Social Sciences: https://forscenter.ch/projects/swiss-household-panel/. Requests to access this dataset should be directed to https://forscenter.ch/projects/swiss-household-panel/.

## Footnotes

^1^Although the expression “political participation” tends to be more strongly associated with actions than attitudes in existing research (Jungkunz and Marx, 2025), throughout this paper, we use the term in a broader sense, referring to behaviors and the attitudinal precursors of these behaviors (Ajzen, 1991). However, when referring solely to attitudinal behavioral dispositions or the specific set of political outcomes we examine, we use the more general and neutral expression “political engagement.”

^2^After 2009, the SHP data includes information on membership in tenants’, women’s, and parents’ associations as an aggregate associational type, which we summarize under the term “local interest groups.” For consistency, we also combined the membership data for these three associations for the years prior to 2009.

^3^Our analyses of trade unions are limited to wage-earners, i.e., the category of individuals on whom unions focus and who are therefore most likely to become members. We use the International Labour Organization’s (ILO) definition of wage-earner, i.e., paid employment in the week prior to the survey interview.

^4^We preferred to focus on membership rather than the more restrictive active involvement in political parties in order to capture a larger share of citizens who are affiliated with political parties. However, the pattern of results we describe below for membership is almost identical to the one for active engagement, albeit with different magnitudes of estimates.

^5^Although an alternative operationalization based on the frequency of voluntary work is possible with the SHP data and yields the same substantive conclusions as described below, we preferred to focus on a binary indicator because it is simpler and more intuitive.

^6^Despite its growing relevance, there is no suitable SHP variable with which to measure digitally networked political participation. This form of participation is hence not part of the dependent variables we consider.

^7^All analyses (except the plots, which were generated in R only) were generated equivalently in R and Stata 18.

^8^CI stands for 95% confidence interval.
