# Supplementary material for: Ascending needles in a haystack? The heterogeneous political participation effects of associational involvement by education
Source: Front Sociol. 2025 Aug 7;10:1584885. doi: 10.3389/fsoc.2025.1584885 (PMC12371539; doi:10.3389/fsoc.2025.1584885)
Supplement: Supplementary file 1 [file Data_Sheet_1.pdf]

Appendices

Appendix A: survey questions and descriptive statistics

Table A1. Survey questions and operationalization of variables

Description	Exact phrasing	Answer modalities (recoded)	Waves
**Sports clubs**	I will now read out a list of associations and organisations. Could you tell me for each of them whether you are an active member, a passive member or not a member? Sports or leisure association	-Nonmember (0)<br>-Passive member<br>-Active member (1)	1999–2009, 2011, 2014, 2017, 2020
**Cultural associations**	Organisation involved in cultural activities, music, or education	-Nonmember (0)<br>-Passive member<br>-Active member (1)	1999–2009, 2011, 2014, 2017, 2020
**Local interest groups**	-1999–2009: Tenants' rights association; Local or parents' association; Women's association<br>-2011, 2014, 2017: Local, parents' or women's association; Other interest groups such as a tenants' rights association, home owners' association, or consumer protection	-Nonmember (0)<br>-Passive member<br>-Active member (1)	1999–2009, 2011, 2014, 2017, 2020
**Trade unions**	Syndicate, employees' association	-Nonmember (0)<br>-Passive member<br>-Active member (1)	1999–2009, 2011, 2014, 2017, 2020
**Environmental organizations**	Organisation concerned with protection of the environment	-Nonmember (0)<br>-Passive member<br>-Active member (1)	1999–2009, 2011, 2014, 2017, 2020
**Charitable associations**	Charitable organisation	-Nonmember (0)<br>-Passive member<br>-Active member (1)	1999–2009, 2011, 2014, 2017, 2020
**Interest in politics**	Generally, how interested are you in politics, if 0 means "not at all interested" and 10 "very interested"?	-0-10	1999-2020
**Feeling of political influence**	How much influence do you think someone like you can have on government policy, if 0 means "no influence", and 10 "a very strong of influence"?	-0-10	1999–2009, 2011, 2014, 2017, 2020
**Participation in federal polls**	Let's suppose that there are 10 federal polls in a year. How many do you usually take part in?	-0-10	1999-2020
**Membership in political party**	I will now read out a list of associations and organisations. Could you tell me for each of them whether you are an active member, a passive member or not a member? Political Party	-Nonmember (0)<br>-Passive or active member (1)	1999–2009, 2011, 2014, 2017, 2020
**Active in voluntary work**	Do you have honorary or voluntary activities within an association, an organisation or an institution? Voluntary activities relating to private initiative, such as helping neighbours, at local fetes are not included here; payments for meetings, expenses or payment of symbolic amounts are not considered as forms of remuneration	-No (0)<br>-Yes (1)	1999-2020

**Propensity to take part in demonstration**	If 0 means "never" and 10 "certainly", tell me to what extent, in the future, you are prepared to: Take part in a demonstration	-0-10	1999-2009
**Propensity to take part in boycott**	If 0 means "never" and 10 "certainly", tell me to what extent, in the future, you are prepared to: Take part in a boycott	-0-10	1999-2009
**Highest level of education achieved**	Constructed	-Incomplete compulsory school; Compulsory school, elementary vocational training; Domestic science course, 1 year school of commerce; General training school (1)<br>-Apprenticeship (CFC, EFZ); Full-time vocational school; Bachelor/maturity; Vocational high school with master certificate, federal certificate; Technical or vocational school (2)<br>-Vocational high school ETS, HTL etc.; University, academic high school, HEP, PH, HES, FH (3)	1999-2020

Table A2. Descriptive statistics on all variables

	Min.	Max.	Mean	Standard deviation	Total observations
Active in sports club	0	1	0.35	0.48	103610
Active in cultural association	0	1	0.17	0.38	102171
Active in local interest group	0	1	0.13	0.34	95226
Active in trade union	0	1	0.06	0.23	101300
Active in environmental organization	0	1	0.03	0.17	94198
Active in charitable association	0	1	0.08	0.28	90365
Interest in politics	0	10	5.48	2.85	111306
Feeling of political influence	0	10	3.84	2.64	104587
Participation in federal polls	0	10	7.72	3.10	93949
Membership in political party	0	1	0.10	0.30	112176
Active in voluntary work	0	1	0.46	0.50	85090
Propensity to take part in demonstration	0	10	3.66	3.57	57570
Propensity to take part in boycott	0	10	3.90	3.68	56996
Primary or less (ref. education)	0	1	0.27	0.44	161305
Secondary	0	1	0.54	0.50	161305
Higher	0	1	0.19	0.39	161305
Man	0	1	0.49	0.50	177116
Age	0	104	42.50	22.25	177017
Foreigner	0	1	0.12	0.33	176816
Lake Geneva (ref. region)	0	1	0.19	0.39	177116
Middleland	0	1	0.25	0.43	177116
North-west	0	1	0.14	0.34	177116
Zurich	0	1	0.16	0.37	177116

East	0	1	0.13	0.34	177116
Center	0	1	0.09	0.29	177116
Ticino	0	1	0.04	0.20	177116
Couple	0	1	0.51	0.50	176990
No children (ref. n. children in HH)	0	1	0.56	0.50	177074
One child	0	1	0.14	0.35	177074
Two children	0	1	0.20	0.40	177074
Three children or more	0	1	0.10	0.30	177074
Full-time (ref. work status)	0	1	0.32	0.47	176487
Part-time	0	1	0.19	0.39	176487
Unemployed	0	1	0.01	0.11	176487
Studying	0	1	0.14	0.35	176487
Non-working	0	1	0.34	0.47	176487
1999 (ref. year)	0	1	0.06	0.24	177116
2000	0	1	0.06	0.23	177116
2001	0	1	0.05	0.22	177116
2002	0	1	0.04	0.21	177116
2003	0	1	0.04	0.19	177116
2004	0	1	0.07	0.25	177116
2005	0	1	0.05	0.22	177116
2006	0	1	0.05	0.22	177116
2007	0	1	0.05	0.22	177116
2008	0	1	0.05	0.22	177116
2009	0	1	0.05	0.22	177116
2011	0	1	0.05	0.22	177116
2014	0	1	0.09	0.28	177116
2017	0	1	0.07	0.25	177116
2020	0	1	0.13	0.33	177116

Table A3. Passive and active membership proportion by associational type and level of education

Membership proportion / Association and education level	Sport			Culture			Local interests			Union			Environment			Charity		
	Prim	Second	Higher	Prim	Second	Higher	Prim	Second	Higher	Prim	Second	Higher	Prim	Second	Higher	Prim	Second	Higher
Passive	4	5.1	5.8	4.3	6.7	9.5	8.2	12.4	15.8	5.4	9.2	10.5	7.2	13.4	21.1	11.2	17.1	19.9
Active	26.1	34	35	10.8	14.8	22.9	8.2	12.2	13.5	2.7	6.1	6.5	1.4	2.5	4.5	4.9	7.5	10.4

Table A4. Active membership propensity by associational type and level of education

Active membership proportion / Association and education level	Sport			Culture			Local interests			Union			Environment			Charity		
	Prim	Second	Higher	Prim	Second	Higher	Prim	Second	Higher	Prim	Second	Higher	Prim	Second	Higher	Prim	Second	Higher
Active membership proportion	86.7	87	85.9	71.5	69	70.7	50	49.6	46.1	33.2	39.7	38.3	16.1	15.8	17.4	30.5	30.3	34.2

Table A5. Pre-membership average in different forms of political participation by associational type and level of education

Political activity dimension / Association and education level	Sport			Culture			Local interests			Union			Environment			Charity		
	Prim	Second	Higher	Prim	Second	Higher	Prim	Second	Higher	Prim	Second	Higher	Prim	Second	Higher	Prim	Second	Higher
Interest in politics	4.2	5.4	6.6	4.6	5.8	7.2	4.4	5.5	6.7	4.4	5.4	6.8	5	5.9	6.9	4.6	5.7	7
Feeling of political influence	2.7	3.4	3.5	2.9	3.5	4	2.4	3.3	3.9	2.6	3.4	4	3.2	3.8	3.7	3	3.6	3.8
Participation in federal polls	6.6	7.3	8.6	7.1	7.9	8.6	6.9	7.6	8.6	7.4	7.5	8.3	7.5	7.9	8.7	7.5	7.9	8.5
Membership in political party	0.006	0.009	0.012	0.007	0.012	0.011	0.003	0.012	0.012	0.006	0.009	0.013	0.009	0.012	0.013	0.006	0.01	0.013
Voluntary work	0.041	0.054	0.059	0.043	0.061	0.059	0.039	0.056	0.056	0.041	0.055	0.062	0.045	0.058	0.053	0.041	0.058	0.057
Propensity to take part in demonstration	2.7	3.6	4.8	2.7	3.6	5	2.7	3.2	4.8	3.7	4.3	5.7	3.6	3.6	4.9	2.6	3.4	4.6

Propensity to take part in boycott	3.1	3.8	4.8	2.8	3.9	5	2.7	3.5	4.8	3	4.3	5.9	3.6	4.1	5.1	2.9	3.8	4.8

Appendix B: regression tables

Table B1. Fixed-effects estimates of the impact of being active in different types of associations on interest in politics interacted with education level

	Sports clubs	Cultural associations	Local interest groups	Trade unions	Environmental organizations	Charitable associations
	estimate<br>standard error<br>p-value<br>95% confidence interval	estimate<br>standard error<br>p-value<br>95% confidence interval	estimate<br>standard error<br>p-value<br>95% confidence interval	estimate<br>standard error<br>p-value<br>95% confidence interval	estimate<br>standard error<br>p-value<br>95% confidence interval	estimate<br>standard error<br>p-value<br>95% confidence interval
Nonmember x Primary or less (ref.)	0	0	0	0	0	0
Active	-0.016	0.092	0.15*	0.33+	-0.056	0.098
	(0.052)	(0.067)	(0.073)	(0.18)	(0.17)	(0.086)
	0.76	0.17	0.039	0.072	0.74	0.25
	-0.12,0.086	-0.039,0.22	0.0074,0.29	-0.029,0.69	-0.38,0.27	-0.071,0.27
Active x Secondary	0.041	-0.089	-0.036	-0.13	0.35+	-0.024
	(0.057)	(0.074)	(0.080)	(0.19)	(0.18)	(0.095)
	0.47	0.23	0.65	0.50	0.051	0.80
	-0.071,0.15	-0.23,0.055	-0.19,0.12	-0.51,0.25	-0.0011,0.71	-0.21,0.16
Active x Higher	-0.030	-0.081	-0.18*	-0.28	0.35+	0.011
	(0.066)	(0.081)	(0.088)	(0.20)	(0.20)	(0.11)
	0.65	0.32	0.036	0.17	0.078	0.92
	-0.16,0.100	-0.24,0.079	-0.36,-0.012	-0.68,0.12	-0.039,0.74	-0.20,0.22
Age	0.038***	0.041***	0.037***	0.035***	0.042***	0.042***
	(0.0046)	(0.0047)	(0.0049)	(0.0091)	(0.0050)	(0.0053)
	6.8e-16	2.0e-18	5.1e-14	0.00011	2.6e-17	2.7e-15
	0.028,0.047	0.032,0.051	0.027,0.047	0.017,0.053	0.032,0.052	0.032,0.053
Age * Age	-0.00019***	-0.00022***	-0.00019***	-0.00015	-0.00024***	-0.00023***
	(0.000042)	(0.000043)	(0.000044)	(0.000098)	(0.000045)	(0.000048)
	0.0000077	0.00000017	0.000022	0.12	0.000000086	0.0000018
	-0.00027,-0.00010	-0.00031,-0.00014	-0.00027,-0.00010	-<br>0.00034,0.000040	-0.00033,-0.00015	-0.00033,-0.00014
Foreigner	0.35***	0.37***	0.31***	0.33**	0.36***	0.33***
	(0.084)	(0.085)	(0.089)	(0.11)	(0.086)	(0.094)
	0.000027	0.000013	0.00053	0.0025	0.000027	0.00056
	0.19,0.52	0.20,0.53	0.13,0.48	0.12,0.55	0.19,0.53	0.14,0.51
Lake Geneva (ref. region)	0	0	0	0	0	0
Middleland	-0.026	0.029	0.037	-0.12	0.088	0.11
	(0.11)	(0.11)	(0.11)	(0.14)	(0.12)	(0.12)
	0.81	0.80	0.74	0.40	0.46	0.37
	-0.24,0.19	-0.19,0.25	-0.18,0.25	-0.39,0.16	-0.15,0.32	-0.13,0.36
North-west	0.048	0.12	0.013	0.018	0.049	0.017
	(0.14)	(0.14)	(0.14)	(0.18)	(0.15)	(0.16)
	0.73	0.40	0.93	0.92	0.75	0.92
	-0.23,0.33	-0.16,0.40	-0.26,0.29	-0.33,0.37	-0.25,0.35	-0.30,0.33
Zurich	0.074	-0.011	-0.035	-0.0042	-0.10	0.016
	(0.14)	(0.15)	(0.14)	(0.18)	(0.16)	(0.17)
	0.61	0.94	0.80	0.98	0.52	0.93
	-0.21,0.35	-0.30,0.28	-0.32,0.24	-0.35,0.35	-0.41,0.21	-0.32,0.35
East	-0.0070	-0.087	-0.069	-0.085	-0.15	-0.021
	(0.15)	(0.16)	(0.16)	(0.20)	(0.17)	(0.18)
	0.96	0.59	0.66	0.68	0.37	0.91
	-0.31,0.30	-0.40,0.22	-0.38,0.24	-0.49,0.32	-0.49,0.18	-0.38,0.33
Center	0.051	0.070	0.059	0.10	-0.015	0.10
	(0.16)	(0.16)	(0.16)	(0.20)	(0.17)	(0.18)
	0.74	0.66	0.70	0.61	0.93	0.56
	-0.26,0.36	-0.24,0.38	-0.25,0.36	-0.29,0.50	-0.34,0.31	-0.25,0.46
Ticino	0.34	0.34	0.42+	0.42	0.15	0.14
	(0.25)	(0.24)	(0.25)	(0.33)	(0.26)	(0.31)
	0.16	0.16	0.089	0.21	0.56	0.66
	-0.14,0.83	-0.14,0.81	-0.064,0.91	-0.23,1.07	-0.36,0.67	-0.48,0.75
Couple	0.064*	0.098***	0.098**	0.13***	0.065*	0.072*
	(0.029)	(0.029)	(0.031)	(0.038)	(0.031)	(0.033)
	0.025	0.00079	0.0013	0.00096	0.037	0.030
	0.0083,0.12	0.041,0.16	0.039,0.16	0.051,0.20	0.0040,0.13	0.0071,0.14

No children (ref. n. children in HH)	0	0	0	0	0	0
One child	0.0064	0.012	-0.017	0.020	0.0078	-0.0082
	(0.025)	(0.025)	(0.026)	(0.031)	(0.027)	(0.028)
	0.80	0.63	0.51	0.52	0.77	0.77
	-0.042,0.055	-0.037,0.061	-0.068,0.034	-0.040,0.080	-0.044,0.060	-0.063,0.047
Two children	-0.037	-0.061*	-0.088**	-0.029	-0.056+	-0.045
	(0.027)	(0.027)	(0.029)	(0.035)	(0.029)	(0.031)
	0.17	0.026	0.0023	0.40	0.056	0.15
	-0.090,0.016	-0.12,-0.0074	-0.14,-0.031	-0.097,0.039	-0.11,0.0014	-0.10,0.016
Three children or more	-0.0012	0.0070	-0.089*	0.059	-0.018	-0.042
	(0.039)	(0.040)	(0.042)	(0.051)	(0.042)	(0.045)
	0.98	0.86	0.035	0.25	0.68	0.35
	-0.078,0.076	-0.071,0.085	-0.17,-0.0062	-0.042,0.16	-0.10,0.065	-0.13,0.047
Full-time (ref. work status)	0	0	0	0	0	0
Part-time	-0.022	-0.022	-0.053+	-0.035	-0.032	-0.031
	(0.027)	(0.027)	(0.029)	(0.035)	(0.030)	(0.032)
	0.41	0.43	0.065	0.32	0.28	0.32
	-0.075,0.030	-0.075,0.032	-0.11,0.0034	-0.10,0.034	-0.090,0.026	-0.093,0.031
Unemployed	0.15*	0.13*	0.12+	0.21	0.11+	0.17*
	(0.060)	(0.061)	(0.063)	(0.14)	(0.064)	(0.067)
	0.012	0.028	0.060	0.14	0.081	0.011
	0.033,0.27	0.014,0.25	-0.0050,0.24	-0.067,0.48	-0.014,0.24	0.040,0.30
Studying	0.15*	0.085	0.16*	0.14	0.068	0.20**
	(0.069)	(0.069)	(0.067)	(0.091)	(0.074)	(0.073)
	0.030	0.22	0.018	0.12	0.36	0.0068
	0.015,0.29	-0.050,0.22	0.028,0.29	-0.037,0.32	-0.076,0.21	0.055,0.34
Non-working	0.089**	0.076**	0.076*	-0.062	0.075*	0.082*
	(0.028)	(0.029)	(0.031)	(0.058)	(0.031)	(0.033)
	0.0018	0.0090	0.012	0.29	0.015	0.014
	0.033,0.14	0.019,0.13	0.017,0.14	-0.18,0.052	0.015,0.14	0.016,0.15
1999 (ref.)	0	0	0	0	0	0
2000	0.20***	0.20***	0.19***	0.24***	0.20***	0.21***
	(0.028)	(0.028)	(0.029)	(0.039)	(0.029)	(0.031)
	6.4e-13	1.3e-12	8.2e-11	9.1e-10	2.6e-11	7.2e-12
	0.14,0.25	0.14,0.26	0.13,0.24	0.16,0.32	0.14,0.25	0.15,0.28
2001	0.32***	0.34***	0.35***	0.33***	0.34***	0.35***
	(0.029)	(0.030)	(0.030)	(0.041)	(0.031)	(0.033)
	1.5e-28	2.1e-30	2.2e-31	1.6e-15	1.3e-28	4.1e-26
	0.26,0.38	0.28,0.40	0.29,0.41	0.25,0.41	0.28,0.40	0.29,0.42
2002	0.31***	0.32***	0.30***	0.33***	0.33***	0.31***
	(0.031)	(0.032)	(0.032)	(0.043)	(0.033)	(0.036)
	1.4e-23	4.0e-24	1.7e-21	1.8e-14	2.7e-24	3.7e-17
	0.25,0.37	0.26,0.38	0.24,0.37	0.24,0.41	0.27,0.40	0.23,0.38
2003	0.30***	0.30***	0.29***	0.29***	0.28***	0.32***
	(0.032)	(0.033)	(0.034)	(0.044)	(0.034)	(0.039)
	9.6e-20	5.0e-20	3.9e-18	8.5e-11	9.5e-17	1.8e-16
	0.23,0.36	0.24,0.37	0.23,0.36	0.20,0.37	0.22,0.35	0.24,0.40
2004	0.48***	0.48***	0.50***	0.50***	0.47***	0.52***
	(0.029)	(0.029)	(0.030)	(0.039)	(0.031)	(0.034)
	8.3e-63	3.5e-61	1.8e-62	1.2e-36	9.2e-54	1.0e-52
	0.42,0.53	0.43,0.54	0.44,0.56	0.42,0.57	0.41,0.53	0.45,0.58
2005	0.45***	0.46***	0.45***	0.46***	0.46***	0.45***
	(0.030)	(0.031)	(0.032)	(0.041)	(0.032)	(0.036)
	2.6e-49	3.6e-50	5.7e-46	4.2e-29	4.4e-45	8.3e-36
	0.39,0.51	0.40,0.52	0.39,0.51	0.38,0.54	0.39,0.52	0.38,0.52
2006	0.31***	0.33***	0.34***	0.35***	0.32***	0.34***
	(0.030)	(0.031)	(0.032)	(0.042)	(0.032)	(0.036)
	9.3e-25	2.2e-26	1.0e-26	2.6e-16	5.0e-23	1.8e-20
	0.25,0.37	0.27,0.39	0.28,0.40	0.26,0.43	0.25,0.38	0.27,0.41
2007	0.31***	0.33***	0.34***	0.32***	0.32***	0.37***
	(0.030)	(0.031)	(0.031)	(0.040)	(0.031)	(0.036)
	5.1e-25	5.6e-27	8.1e-28	3.3e-15	2.2e-24	4.4e-25
	0.25,0.37	0.27,0.39	0.28,0.40	0.24,0.39	0.26,0.38	0.30,0.44
2008	0.44***	0.44***	0.46***	0.46***	0.45***	0.47***
	(0.030)	(0.031)	(0.032)	(0.040)	(0.032)	(0.037)
	6.2e-48	2.8e-46	1.6e-47	1.1e-29	5.1e-45	5.2e-38
	0.38,0.50	0.38,0.50	0.40,0.52	0.38,0.53	0.39,0.51	0.40,0.55
2009	0.40***	0.43***	0.41***	0.42***	0.44***	0.46***
	(0.030)	(0.031)	(0.032)	(0.040)	(0.031)	(0.037)

	5.8e-40	1.3e-44	5.9e-38	7.9e-26	1.6e-43	1.0e-35
	0.34,0.46	0.37,0.49	0.35,0.47	0.34,0.50	0.37,0.50	0.39,0.53
2011	-0.12***	-0.11***	-0.087**	-0.14***	-0.12***	-0.10***
	(0.026)	(0.026)	(0.028)	(0.037)	(0.028)	(0.029)
	0.0000072	0.000037	0.0019	0.00016	0.000036	0.00049
	-0.17,-0.065	-0.16,-0.057	-0.14,-0.032	-0.21,-0.067	-0.17,-0.061	-0.16,-0.044
2014	-0.089***	-0.071**	-0.058*	-0.098**	-0.062*	-0.081**
	(0.024)	(0.024)	(0.026)	(0.034)	(0.026)	(0.027)
	0.00016	0.0030	0.023	0.0040	0.015	0.0024
	-0.13,-0.043	-0.12,-0.024	-0.11,-0.0079	-0.16,-0.031	-0.11,-0.012	-0.13,-0.029
2017	-0.28***	-0.27***	-0.25***	-0.34***	-0.26***	-0.27***
	(0.025)	(0.025)	(0.027)	(0.036)	(0.027)	(0.028)
	3.3e-30	3.4e-27	2.3e-21	2.3e-21	4.8e-23	1.4e-21
	-0.33,-0.23	-0.32,-0.22	-0.31,-0.20	-0.41,-0.27	-0.32,-0.21	-0.32,-0.21
2020	-0.24***	-0.25***	-0.24***	-0.28***	-0.26***	-0.25***
	(0.022)	(0.023)	(0.024)	(0.032)	(0.024)	(0.025)
	7.1e-27	3.2e-28	4.7e-24	4.4e-18	1.7e-26	1.7e-22
	-0.28,-0.20	-0.29,-0.21	-0.29,-0.20	-0.34,-0.22	-0.31,-0.21	-0.30,-0.20
Constant	3.81***	3.59***	3.77***	3.76***	3.56***	3.48***
	(0.16)	(0.16)	(0.16)	(0.24)	(0.17)	(0.18)
	1.7e-125	6.1e-110	2.8e-118	1.0e-55	1.5e-95	4.8e-84
	3.50,4.12	3.28,3.91	3.45,4.09	3.29,4.22	3.23,3.90	3.13,3.83
Individuals	28060	28089	27373	17672	26896	26716
Observations	90653	89846	84398	47745	85164	76218

Note: significance levels: + p < 0.10. * p < 0.05. ** p < 0.01. *** p < 0.001. Source: Swiss Household Panel (SHP)

Table B2. Fixed-effects estimates of the impact of being active in different types of associations on the feeling of political influence interacted with education level

	Sports clubs<br>estimate<br>standard error<br>p-value<br>95% confidence interval	Cultural associations<br>estimate<br>standard error<br>p-value<br>95% confidence interval	Local interest groups<br>estimate<br>standard error<br>p-value<br>95% confidence interval	Trade unions<br>estimate<br>standard error<br>p-value<br>95% confidence interval	Environmental organizations<br>estimate<br>standard error<br>p-value<br>95% confidence interval	Charitable associations<br>estimate<br>standard error<br>p-value<br>95% confidence interval
Nonmember x Primary or less (ref.)	0	0	0	0	0	0
Active	0.035	0.083	0.14	0.72**	0.38	-0.024
	(0.074)	(0.093)	(0.10)	(0.26)	(0.23)	(0.12)
	0.64	0.37	0.16	0.0053	0.11	0.84
	-0.11,0.18	-0.098,0.26	-0.056,0.34	0.21,1.22	-0.079,0.83	-0.26,0.21
Active x Secondary	0.0057	0.033	-0.078	-0.51+	-0.096	0.18
	(0.082)	(0.10)	(0.11)	(0.27)	(0.25)	(0.13)
	0.94	0.75	0.48	0.059	0.70	0.17
	-0.15,0.17	-0.17,0.23	-0.30,0.14	-1.05,0.019	-0.59,0.40	-0.079,0.44
Active x Higher	0.16+	0.072	-0.011	-0.60*	-0.018	0.20
	(0.094)	(0.11)	(0.12)	(0.29)	(0.28)	(0.15)
	0.097	0.52	0.93	0.037	0.95	0.19
	-0.028,0.34	-0.15,0.29	-0.25,0.23	-1.16,-0.036	-0.56,0.53	-0.097,0.49
Age	0.068***	0.065***	0.066***	0.088***	0.071***	0.064***
	(0.0065)	(0.0065)	(0.0068)	(0.013)	(0.0068)	(0.0073)
	3.2e-26	1.1e-23	4.6e-22	6.3e-12	2.5e-25	1.6e-18
	0.056,0.081	0.053,0.078	0.053,0.079	0.063,0.11	0.058,0.085	0.050,0.078
Age * Age	-0.00023***	-0.00019**	-0.00020**	-0.00041**	-0.00024***	-0.00018**
	(0.000058)	(0.000059)	(0.000061)	(0.00014)	(0.000061)	(0.000066)
	0.000088	0.0012	0.0011	0.0028	0.000063	0.0070
	-0.00034,-0.00011	-0.00031,-0.000075	-0.00032,-0.000080	-0.00067,-0.00014	-0.00036,-0.00012	-0.00031,-0.000049
Foreigner	-0.22+	-0.31**	-0.31*	-0.42**	-0.26*	-0.28*
	(0.12)	(0.12)	(0.13)	(0.16)	(0.12)	(0.13)
	0.068	0.0086	0.013	0.0084	0.034	0.036
	-0.45,0.016	-0.55,-0.080	-0.56,-0.064	-0.73,-0.11	-0.50,-0.019	-0.54,-0.018
Lake Geneva (ref. region)	0	0	0	0	0	0
Middleland	0.057	-0.034	0.091	-0.068	0.062	0.048
	(0.15)	(0.15)	(0.15)	(0.20)	(0.16)	(0.17)
	0.70	0.82	0.55	0.73	0.70	0.78
	-0.24,0.35	-0.34,0.27	-0.21,0.39	-0.45,0.32	-0.26,0.38	-0.29,0.38
North-west	0.016	0.027	-0.038	0.18	-0.064	0.20
	(0.20)	(0.20)	(0.19)	(0.25)	(0.21)	(0.22)
	0.93	0.89	0.84	0.49	0.76	0.36
	-0.37,0.40	-0.36,0.41	-0.42,0.34	-0.32,0.67	-0.48,0.35	-0.23,0.64
Zurich	0.058	-0.14	-0.052	-0.25	0.017	0.034
	(0.20)	(0.20)	(0.20)	(0.25)	(0.22)	(0.23)
	0.77	0.49	0.79	0.31	0.94	0.88
	-0.33,0.45	-0.54,0.26	-0.44,0.34	-0.74,0.24	-0.41,0.44	-0.43,0.49
East	0.075	-0.11	-0.091	0.047	0.13	0.16
	(0.22)	(0.22)	(0.22)	(0.29)	(0.24)	(0.25)
	0.73	0.62	0.68	0.87	0.60	0.52
	-0.35,0.50	-0.54,0.32	-0.52,0.34	-0.53,0.62	-0.34,0.59	-0.33,0.66
Center	0.32	0.12	0.25	0.16	0.26	0.36
	(0.22)	(0.22)	(0.22)	(0.28)	(0.23)	(0.25)
	0.15	0.58	0.25	0.57	0.26	0.14
	-0.11,0.74	-0.31,0.55	-0.17,0.67	-0.39,0.72	-0.19,0.71	-0.12,0.84
Ticino	-0.38	-0.26	-0.31	-0.51	-0.49	-0.97*
	(0.35)	(0.34)	(0.35)	(0.47)	(0.37)	(0.45)
	0.27	0.44	0.38	0.27	0.18	0.029
	-1.07,0.30	-0.92,0.40	-1.00,0.38	-1.43,0.40	-1.21,0.22	-1.85,-0.100
Couple	-0.0071	-0.0013	0.00034	0.065	0.0033	-0.010
	(0.040)	(0.040)	(0.042)	(0.054)	(0.042)	(0.045)
	0.86	0.97	0.99	0.23	0.94	0.82
	-0.085,0.071	-0.080,0.077	-0.082,0.083	-0.040,0.17	-0.080,0.086	-0.099,0.079
No children (ref. n. children in HH)	0	0	0	0	0	0

	0,0	0,0	0,0	0,0	0,0	0,0
One child	0.042	0.055	0.019	0.029	0.035	0.037
	(0.034)	(0.035)	(0.036)	(0.043)	(0.036)	(0.039)
	0.22	0.11	0.59	0.51	0.34	0.33
	-0.025,0.11	-0.013,0.12	-0.052,0.090	-0.056,0.11	-0.037,0.11	-0.038,0.11
Two children	-0.00071	-0.032	-0.080*	-0.080+	-0.060	-0.018
	(0.038)	(0.038)	(0.040)	(0.049)	(0.040)	(0.042)
	0.99	0.39	0.047	0.098	0.14	0.67
	-0.075,0.073	-0.11,0.042	-0.16,-0.0012	-0.18,0.015	-0.14,0.019	-0.10,0.064
Three children or more	0.098+	0.071	0.015	0.096	0.033	0.00038
	(0.054)	(0.055)	(0.058)	(0.072)	(0.058)	(0.062)
	0.072	0.19	0.80	0.18	0.56	1.00
	-0.0089,0.20	-0.036,0.18	-0.100,0.13	-0.045,0.24	-0.080,0.15	-0.12,0.12
Full-time (ref. work status)	0	0	0	0	0	0
Part-time	-0.075*	-0.075*	-0.10**	-0.085+	-0.079+	-0.080+
	(0.037)	(0.038)	(0.040)	(0.050)	(0.041)	(0.044)
	0.044	0.047	0.0094	0.089	0.051	0.067
	-0.15,-0.0022	-0.15,-0.00091	-0.18,-0.026	-0.18,0.013	-0.16,0.00048	-0.17,0.0055
Unemployed	-0.014	0.0012	-0.029	-0.022	0.0075	0.094
	(0.085)	(0.085)	(0.088)	(0.20)	(0.089)	(0.094)
	0.87	0.99	0.74	0.91	0.93	0.31
	-0.18,0.15	-0.17,0.17	-0.20,0.14	-0.41,0.37	-0.17,0.18	-0.089,0.28
Studying	-0.064	0.021	0.033	0.072	-0.045	-0.042
	(0.098)	(0.097)	(0.095)	(0.13)	(0.10)	(0.10)
	0.51	0.83	0.73	0.58	0.67	0.68
	-0.26,0.13	-0.17,0.21	-0.15,0.22	-0.18,0.33	-0.25,0.16	-0.24,0.16
Non-working	-0.069+	-0.062	-0.093*	-0.22**	-0.066	-0.077+
	(0.040)	(0.040)	(0.043)	(0.082)	(0.042)	(0.046)
	0.081	0.12	0.028	0.0059	0.12	0.092
	-0.15,0.0085	-0.14,0.017	-0.18,-0.0100	-0.38,-0.065	-0.15,0.017	-0.17,0.013
1999 (ref.)	0	0	0	0	0	0
2000	0.23***	0.22***	0.23***	0.24***	0.23***	0.21***
	(0.038)	(0.039)	(0.039)	(0.055)	(0.040)	(0.042)
	2.1e-09	0.000000016	5.1e-09	0.000010	6.2e-09	0.0000012
	0.15,0.30	0.14,0.29	0.15,0.31	0.13,0.35	0.15,0.31	0.12,0.29
2001	0.26***	0.25***	0.24***	0.34***	0.26***	0.21***
	(0.040)	(0.040)	(0.041)	(0.057)	(0.042)	(0.045)
	6.1e-11	1.1e-09	4.6e-09	2.5e-09	5.6e-10	0.0000020
	0.18,0.34	0.17,0.32	0.16,0.32	0.23,0.45	0.18,0.34	0.13,0.30
2002	0.28***	0.27***	0.25***	0.30***	0.26***	0.21***
	(0.043)	(0.043)	(0.044)	(0.059)	(0.044)	(0.049)
	3.2e-11	3.0e-10	0.000000013	0.00000047	4.9e-09	0.000013
	0.20,0.37	0.19,0.35	0.16,0.34	0.18,0.41	0.17,0.35	0.12,0.31
2003	0.11*	0.14**	0.16***	0.24***	0.12*	0.052
	(0.045)	(0.045)	(0.046)	(0.061)	(0.046)	(0.052)
	0.012	0.0017	0.00044	0.000081	0.012	0.32
	0.025,0.20	0.052,0.23	0.072,0.25	0.12,0.36	0.026,0.21	-0.051,0.15
2004	0.20***	0.23***	0.24***	0.30***	0.23***	0.22***
	(0.039)	(0.040)	(0.041)	(0.054)	(0.041)	(0.046)
	0.00000041	4.9e-09	8.9e-09	0.000000029	0.000000036	0.0000012
	0.12,0.27	0.15,0.31	0.16,0.32	0.19,0.41	0.15,0.31	0.13,0.31
2005	0.14***	0.15***	0.17***	0.28***	0.17***	0.13**
	(0.041)	(0.042)	(0.043)	(0.056)	(0.044)	(0.049)
	0.00058	0.00032	0.000059	0.00000087	0.00015	0.0070
	0.061,0.22	0.069,0.23	0.089,0.26	0.17,0.39	0.080,0.25	0.036,0.23
2006	0.20***	0.24***	0.26***	0.30***	0.27***	0.22***
	(0.041)	(0.042)	(0.043)	(0.058)	(0.043)	(0.049)
	0.00000095	8.9e-09	1.1e-09	0.00000019	6.3e-10	0.0000053
	0.12,0.28	0.16,0.32	0.18,0.35	0.19,0.42	0.18,0.35	0.13,0.32
2007	0.17***	0.21***	0.22***	0.33***	0.21***	0.22***
	(0.041)	(0.041)	(0.043)	(0.055)	(0.043)	(0.048)
	0.000047	0.00000033	0.00000032	2.2e-09	0.00000049	0.0000038
	0.087,0.25	0.13,0.29	0.13,0.30	0.22,0.44	0.13,0.30	0.13,0.32
2008	0.19***	0.17***	0.19***	0.30***	0.16***	0.19***
	(0.041)	(0.042)	(0.043)	(0.056)	(0.043)	(0.050)
	0.0000046	0.000056	0.000016	0.000000056	0.00030	0.00016
	0.11,0.27	0.087,0.25	0.10,0.27	0.19,0.41	0.071,0.24	0.090,0.28
2009	0.095*	0.11*	0.087*	0.21***	0.10*	0.079
	(0.042)	(0.042)	(0.043)	(0.055)	(0.043)	(0.049)
	0.022	0.010	0.045	0.00014	0.017	0.11

	0.014,0.18	0.026,0.19	0.0019,0.17	0.10,0.32	0.018,0.18	-0.018,0.18
2011	0.12**	0.12***	0.12**	0.22***	0.14***	0.12**
	(0.035)	(0.036)	(0.038)	(0.051)	(0.038)	(0.039)
	0.0011	0.00054	0.0012	0.000019	0.00018	0.0020
	0.046,0.18	0.053,0.19	0.049,0.20	0.12,0.32	0.067,0.22	0.044,0.20
2014	0.056	0.070+	0.080*	0.072	0.082*	0.055
	(0.036)	(0.037)	(0.040)	(0.053)	(0.039)	(0.041)
	0.12	0.056	0.043	0.18	0.037	0.17
	-0.015,0.13	-0.0019,0.14	0.0026,0.16	-0.032,0.18	0.0050,0.16	-0.025,0.14
2017	-0.0049	0.015	0.022	-0.012	0.015	-0.0023
	(0.034)	(0.035)	(0.037)	(0.050)	(0.037)	(0.038)
	0.89	0.67	0.55	0.81	0.69	0.95
	-0.072,0.062	-0.053,0.083	-0.051,0.095	-0.11,0.086	-0.057,0.087	-0.078,0.073
2020	0.096**	0.11***	0.100**	0.044	0.079*	0.089**
	(0.031)	(0.031)	(0.033)	(0.045)	(0.033)	(0.034)
	0.0019	0.00044	0.0027	0.33	0.017	0.0096
	0.035,0.16	0.048,0.17	0.035,0.16	-0.044,0.13	0.014,0.14	0.022,0.16
Constant	0.89***	1.02***	1.04***	0.86**	0.75**	1.00***
	(0.22)	(0.22)	(0.23)	(0.33)	(0.24)	(0.24)
	0.000058	0.0000043	0.0000046	0.0098	0.0014	0.000044
	0.46,1.32	0.58,1.45	0.59,1.48	0.21,1.52	0.29,1.21	0.52,1.48
Individuals	25767	25788	25173	16274	24627	24517
Observations	84605	83805	78859	44680	79336	70849

Note: significance levels: + p < 0.10. * p < 0.05. ** p < 0.01. *** p < 0.001. Source: Swiss Household Panel (SHP)

Table B3. Fixed-effects estimates of the impact of being active in different types of associations on participation in federal polls interacted with education level

	Sports clubs<br><br>estimate<br>standard error<br>p-value<br>95% confidence interval	Cultural associations<br><br>estimate<br>standard error<br>p-value<br>95% confidence interval	Local interest groups<br><br>estimate<br>standard error<br>p-value<br>95% confidence interval	Trade unions<br><br>estimate<br>standard error<br>p-value<br>95% confidence interval	Environmental organizations<br><br>estimate<br>standard error<br>p-value<br>95% confidence interval	Charitable associations<br><br>estimate<br>standard error<br>p-value<br>95% confidence interval
Nonmember x Primary or less (ref.)	0	0	0	0	0	0
Active	0.23**	0.045	-0.032	-0.32	-0.23	0.071
	(0.070)	(0.090)	(0.092)	(0.26)	(0.23)	(0.11)
	0.0012	0.62	0.73	0.21	0.31	0.52
	0.089,0.36	-0.13,0.22	-0.21,0.15	-0.83,0.18	-0.67,0.21	-0.15,0.29
Active x Secondary	-0.17*	0.037	0.15	0.45+	0.35	-0.022
	(0.076)	(0.097)	(0.100)	(0.27)	(0.24)	(0.12)
	0.022	0.70	0.12	0.094	0.14	0.85
	-0.32,-0.025	-0.15,0.23	-0.042,0.35	-0.077,0.98	-0.12,0.83	-0.26,0.22
Active x Higher	-0.22*	-0.11	0.010	0.46+	0.36	0.032
	(0.086)	(0.11)	(0.11)	(0.28)	(0.26)	(0.14)
	0.012	0.29	0.93	0.099	0.17	0.82
	-0.38,-0.048	-0.32,0.096	-0.21,0.23	-0.088,1.01	-0.16,0.88	-0.24,0.30
Age	0.072***	0.078***	0.068***	0.046***	0.077***	0.079***
	(0.0057)	(0.0059)	(0.0061)	(0.012)	(0.0062)	(0.0067)
	1.2e-36	3.8e-40	1.8e-28	0.000083	4.6e-35	1.2e-31
	0.061,0.084	0.067,0.090	0.056,0.080	0.023,0.069	0.065,0.089	0.066,0.092
Age * Age	-0.00040***	-0.00044***	-0.00035***	-0.000072	-0.00043***	-0.00045***
	(0.000051)	(0.000053)	(0.000055)	(0.00012)	(0.000055)	(0.000061)
	4.3e-15	1.4e-16	1.7e-10	0.56	4.6e-15	1.3e-13
	-0.00050,-0.00030	-0.00054,-0.00033	-0.00046,-0.00024	-0.00032,0.00017	-0.00054,-0.00033	-0.00057,-0.00033
Foreigner	0.61***	0.47**	0.68***	-0.077	0.47**	0.33+
	(0.16)	(0.16)	(0.17)	(0.23)	(0.16)	(0.19)
	0.000076	0.0032	0.000061	0.74	0.0036	0.078
	0.31,0.92	0.16,0.78	0.35,1.02	-0.53,0.37	0.15,0.79	-0.036,0.69
Lake Geneva (ref. region)	0	0	0	0	0	0
Middleland	-0.27*	-0.18	-0.18	-0.16	-0.13	-0.012
	(0.13)	(0.14)	(0.14)	(0.18)	(0.15)	(0.16)
	0.045	0.20	0.17	0.36	0.37	0.94
	-0.53,-0.0054	-0.45,0.093	-0.45,0.081	-0.51,0.19	-0.42,0.16	-0.32,0.30
North-west	-0.24	-0.14	-0.18	-0.74**	-0.20	-0.076
	(0.17)	(0.18)	(0.17)	(0.23)	(0.19)	(0.20)
	0.18	0.43	0.29	0.0014	0.28	0.71
	-0.58,0.10	-0.48,0.21	-0.52,0.16	-1.20,-0.29	-0.57,0.17	-0.48,0.32
Zurich	0.15	0.081	0.023	0.064	0.090	0.24
	(0.17)	(0.18)	(0.18)	(0.23)	(0.19)	(0.21)
	0.40	0.66	0.90	0.78	0.64	0.25
	-0.20,0.49	-0.28,0.44	-0.32,0.37	-0.39,0.52	-0.29,0.47	-0.17,0.66
East	0.32+	0.29	0.13	0.092	0.047	0.50*
	(0.19)	(0.20)	(0.20)	(0.27)	(0.22)	(0.23)
	0.090	0.14	0.51	0.73	0.83	0.031
	-0.051,0.70	-0.099,0.68	-0.26,0.51	-0.44,0.62	-0.38,0.47	0.045,0.95
Center	0.0030	0.041	-0.029	-0.032	-0.075	0.13
	(0.19)	(0.20)	(0.19)	(0.26)	(0.21)	(0.22)
	0.99	0.83	0.88	0.90	0.72	0.57
	-0.37,0.38	-0.34,0.43	-0.41,0.35	-0.53,0.47	-0.48,0.33	-0.31,0.57
Ticino	-0.42	-0.43	-0.20	-0.36	-0.32	-0.16
	(0.31)	(0.30)	(0.31)	(0.43)	(0.33)	(0.39)
	0.17	0.15	0.53	0.40	0.33	0.68
	-1.04,0.19	-1.02,0.16	-0.80,0.41	-1.19,0.47	-0.97,0.33	-0.93,0.61
Couple	0.37***	0.37***	0.39***	0.35***	0.35***	0.33***
	(0.035)	(0.035)	(0.037)	(0.048)	(0.038)	(0.041)
	5.0e-27	4.5e-25	1.9e-25	7.5e-13	9.3e-20	5.7e-16
	0.31,0.44	0.30,0.44	0.32,0.46	0.25,0.44	0.27,0.42	0.25,0.41
No children (ref. n. children in HH)	0	0	0	0	0	0
One child	-0.12***	-0.13***	-0.13***	-0.096*	-0.17***	-0.17***

	(0.031)	(0.032)	(0.033)	(0.040)	(0.034)	(0.036)
	0.000084	0.000041	0.000100	0.016	0.0000010	0.0000024
	-0.18,-0.061	-0.19,-0.068	-0.19,-0.064	-0.17,-0.018	-0.23,-0.100	-0.24,-0.10
Two children	-0.079*	-0.080*	-0.097**	-0.040	-0.082*	-0.098*
	(0.034)	(0.034)	(0.036)	(0.044)	(0.037)	(0.039)
	0.018	0.021	0.0080	0.36	0.027	0.012
	-0.15,-0.013	-0.15,-0.012	-0.17,-0.025	-0.13,0.047	-0.15,-0.0091	-0.18,-0.021
Three children or more	-0.098*	-0.077	-0.070	-0.033	-0.11*	-0.084
	(0.048)	(0.050)	(0.053)	(0.065)	(0.053)	(0.058)
	0.042	0.12	0.19	0.61	0.031	0.15
	-0.19,-0.0035	-0.17,0.021	-0.17,0.034	-0.16,0.095	-0.22,-0.011	-0.20,0.030
Full-time (ref. work status)	0	0	0	0	0	0
Part-time	0.077*	0.082*	0.067+	0.10*	0.071+	0.074+
	(0.033)	(0.034)	(0.035)	(0.045)	(0.037)	(0.039)
	0.018	0.016	0.059	0.020	0.052	0.059
	0.013,0.14	0.016,0.15	-0.0025,0.14	0.017,0.19	-0.00076,0.14	-0.0029,0.15
Unemployed	0.054	0.10	0.084	-0.24	0.060	0.13
	(0.079)	(0.080)	(0.083)	(0.19)	(0.085)	(0.090)
	0.49	0.20	0.31	0.22	0.48	0.15
	-0.10,0.21	-0.053,0.26	-0.078,0.25	-0.61,0.14	-0.11,0.23	-0.046,0.31
Studying	0.37***	0.44***	0.33***	0.35**	0.44***	0.36***
	(0.098)	(0.099)	(0.095)	(0.12)	(0.11)	(0.11)
	0.00015	0.0000084	0.00047	0.0047	0.000032	0.00068
	0.18,0.56	0.25,0.63	0.15,0.52	0.11,0.60	0.23,0.65	0.15,0.57
Non-working	0.23***	0.23***	0.19***	0.045	0.24***	0.22***
	(0.035)	(0.036)	(0.038)	(0.072)	(0.038)	(0.042)
	5.3e-11	1.1e-10	0.00000055	0.53	6.3e-10	0.00000013
	0.16,0.29	0.16,0.30	0.11,0.26	-0.096,0.19	0.16,0.31	0.14,0.30
1999 (ref.)	0	0	0	0	0	0
2000	0.24***	0.21***	0.23***	0.22***	0.21***	0.23***
	(0.034)	(0.035)	(0.035)	(0.050)	(0.036)	(0.039)
	5.9e-13	2.8e-09	3.6e-11	0.0000085	0.000000015	3.6e-09
	0.18,0.31	0.14,0.27	0.16,0.30	0.12,0.32	0.13,0.28	0.15,0.31
2001	0.18***	0.18***	0.20***	0.19***	0.23***	0.25***
	(0.035)	(0.036)	(0.037)	(0.052)	(0.038)	(0.042)
	0.00000038	0.00000057	0.000000055	0.00023	8.3e-10	2.5e-09
	0.11,0.25	0.11,0.25	0.13,0.27	0.090,0.29	0.16,0.31	0.17,0.33
2002	0.33***	0.38***	0.35***	0.45***	0.41***	0.39***
	(0.038)	(0.039)	(0.039)	(0.054)	(0.040)	(0.045)
	1.3e-18	3.3e-22	1.6e-18	4.1e-17	4.4e-24	3.5e-18
	0.26,0.40	0.30,0.45	0.27,0.42	0.35,0.56	0.33,0.49	0.31,0.48
2003	0.13**	0.17***	0.14***	0.19***	0.15***	0.16***
	(0.039)	(0.040)	(0.042)	(0.055)	(0.042)	(0.049)
	0.0010	0.000031	0.00086	0.00063	0.00045	0.00091
	0.052,0.21	0.089,0.25	0.057,0.22	0.080,0.30	0.065,0.23	0.066,0.26
2004	0.42***	0.49***	0.45***	0.56***	0.47***	0.48***
	(0.035)	(0.036)	(0.037)	(0.049)	(0.038)	(0.042)
	1.6e-34	2.7e-43	7.7e-35	3.0e-30	2.5e-35	2.3e-30
	0.36,0.49	0.42,0.56	0.38,0.52	0.46,0.66	0.39,0.54	0.40,0.56
2005	0.46***	0.50***	0.49***	0.55***	0.51***	0.55***
	(0.037)	(0.038)	(0.039)	(0.051)	(0.040)	(0.045)
	2.0e-36	1.5e-39	2.2e-36	4.9e-27	2.4e-37	7.6e-34
	0.39,0.53	0.42,0.57	0.41,0.56	0.45,0.65	0.43,0.59	0.46,0.63
2006	0.26***	0.28***	0.29***	0.34***	0.28***	0.31***
	(0.037)	(0.038)	(0.039)	(0.053)	(0.039)	(0.045)
	1.3e-12	1.2e-13	1.1e-13	1.7e-10	6.4e-13	5.8e-12
	0.19,0.33	0.21,0.35	0.21,0.36	0.23,0.44	0.21,0.36	0.22,0.40
2007	0.14***	0.15***	0.16***	0.19***	0.12**	0.15***
	(0.036)	(0.037)	(0.038)	(0.050)	(0.039)	(0.045)
	0.00014	0.000061	0.000046	0.00021	0.0013	0.00078
	0.067,0.21	0.076,0.22	0.081,0.23	0.088,0.28	0.048,0.20	0.062,0.24
2008	0.11**	0.17***	0.16***	0.19***	0.13**	0.14**
	(0.037)	(0.038)	(0.039)	(0.050)	(0.039)	(0.046)
	0.0035	0.0000071	0.000028	0.00017	0.0011	0.0022
	0.035,0.18	0.095,0.24	0.087,0.24	0.091,0.29	0.051,0.20	0.050,0.23
2009	0.17***	0.22***	0.20***	0.19***	0.18***	0.20***
	(0.036)	(0.037)	(0.038)	(0.050)	(0.038)	(0.045)
	0.0000034	6.2e-09	0.00000026	0.00021	0.0000021	0.0000082
	0.098,0.24	0.14,0.29	0.12,0.27	0.087,0.28	0.11,0.26	0.11,0.29
2011	-0.020	-0.0013	-0.029	0.067	0.016	0.013

	(0.031)	(0.032)	(0.034)	(0.046)	(0.034)	(0.035)
	0.52	0.97	0.39	0.14	0.64	0.70
	-0.080,0.040	-0.063,0.061	-0.096,0.037	-0.023,0.16	-0.050,0.082	-0.056,0.083
2014	0.17***	0.18***	0.17***	0.22***	0.21***	0.20***
	(0.032)	(0.032)	(0.035)	(0.047)	(0.035)	(0.037)
	0.00000010	0.000000035	0.00000065	0.0000035	2.3e-09	0.000000023
	0.11,0.23	0.12,0.24	0.10,0.24	0.13,0.31	0.14,0.28	0.13,0.28
2017	0.13***	0.16***	0.16***	0.13**	0.16***	0.16***
	(0.030)	(0.031)	(0.033)	(0.045)	(0.033)	(0.034)
	0.000023	0.00000021	0.00000072	0.0042	0.00000076	0.0000027
	0.067,0.18	0.099,0.22	0.098,0.23	0.040,0.22	0.098,0.23	0.094,0.23
2020	0.15***	0.15***	0.16***	0.15***	0.16***	0.18***
	(0.027)	(0.027)	(0.029)	(0.040)	(0.029)	(0.031)
	0.000000036	0.000000048	0.000000060	0.00020	0.000000020	6.3e-09
	0.095,0.20	0.096,0.20	0.10,0.22	0.071,0.23	0.11,0.22	0.12,0.24
Constant	4.65***	4.36***	4.70***	5.18***	4.36***	4.20***
	(0.20)	(0.20)	(0.20)	(0.31)	(0.22)	(0.23)
	4.8e-122	7.9e-104	4.3e-117	1.3e-63	6.3e-91	3.4e-76
	4.27,5.04	3.96,4.75	4.30,5.10	4.58,5.79	3.94,4.78	3.76,4.65
Individuals	22265	22307	21739	14118	21184	21086
Observations	75780	74782	69916	39664	70235	62282

Note: significance levels: + p < 0.10. * p < 0.05. ** p < 0.01. *** p < 0.001. Source: Swiss Household Panel (SHP)

Table B4. Fixed-effects estimates of the impact of being active in different types of associations on membership in political party interacted with education level

	Sports clubs<br><br>estimate<br>standard error<br>p-value<br>95% confidence interval	Cultural associations<br><br>estimate<br>standard error<br>p-value<br>95% confidence interval	Local interest groups<br><br>estimate<br>standard error<br>p-value<br>95% confidence interval	Trade unions<br><br>estimate<br>standard error<br>p-value<br>95% confidence interval	Environmental organizations<br><br>estimate<br>standard error<br>p-value<br>95% confidence interval	Charitable associations<br><br>estimate<br>standard error<br>p-value<br>95% confidence interval
Nonmember x Primary or less (ref.)	0	0	0	0	0	0
Active	0.024***	0.046***	0.052***	0.025	0.11***	0.055***
	(0.0063)	(0.0076)	(0.0087)	(0.021)	(0.018)	(0.0094)
	0.00013	9.8e-10	2.1e-09	0.22	1.4e-09	4.6e-09
	0.012,0.036	0.032,0.061	0.035,0.069	-0.015,0.066	0.072,0.14	0.037,0.074
Active x Secondary	-0.0021	-0.024**	-0.026**	0.028	0.0070	-0.020+
	(0.0070)	(0.0084)	(0.0095)	(0.022)	(0.019)	(0.010)
	0.77	0.0042	0.0066	0.21	0.72	0.051
	-0.016,0.012	-0.041,-0.0076	-0.044,-0.0072	-0.015,0.071	-0.031,0.045	-0.041,0.000066
Active x Higher	-0.016*	-0.038***	-0.052***	0.028	-0.041+	-0.060***
	(0.0081)	(0.0094)	(0.011)	(0.023)	(0.021)	(0.012)
	0.043	0.000064	0.00000067	0.22	0.059	0.00000063
	-0.032,-0.00052	-0.056,-0.019	-0.073,-0.032	-0.017,0.073	-0.083,0.0016	-0.083,-0.036
Age	0.0047***	0.0039***	0.0028***	0.0029**	0.0038***	0.0033***
	(0.00058)	(0.00056)	(0.00059)	(0.0010)	(0.00055)	(0.00059)
	5.2e-16	3.5e-12	0.0000016	0.0048	6.1e-12	0.000000030
	0.0036,0.0059	0.0028,0.0050	0.0017,0.0040	0.00089,0.0049	0.0027,0.0049	0.0021,0.0044
Age * Age	-0.000039***	-0.000033***	-0.000022***	-0.000024*	-0.000035***	-0.000027***
	(0.0000052)	(0.0000050)	(0.0000053)	(0.000011)	(0.0000050)	(0.0000053)
	9.6e-14	1.0e-10	0.000024	0.030	2.7e-12	0.00000040
	-0.000049,-	-0.000042,-	-0.000033,-	-0.000046,-	-0.000044,-	-0.000037,-
	0.000029	0.000023	0.000012	0.0000024	0.000025	0.000017
Foreigner	0.018+	0.012	0.0084	0.014	0.014	0.0071
	(0.011)	(0.0099)	(0.011)	(0.012)	(0.0096)	(0.010)
	0.085	0.21	0.43	0.26	0.14	0.50
	-0.0025,0.039	-0.0071,0.032	-0.012,0.029	-0.010,0.039	-0.0047,0.033	-0.013,0.027
Lake Geneva (ref. region)	0	0	0	0	0	0
Middleland	-0.018	-0.0047	0.0053	-0.017	-0.022+	-0.020
	(0.014)	(0.013)	(0.013)	(0.016)	(0.013)	(0.014)
	0.18	0.72	0.69	0.30	0.096	0.15
	-0.045,0.0086	-0.031,0.021	-0.021,0.031	-0.048,0.015	-0.048,0.0039	-0.047,0.0072
North-west	-0.024	0.020	0.0095	0.010	-0.032+	-0.011
	(0.018)	(0.017)	(0.017)	(0.021)	(0.017)	(0.018)
	0.17	0.23	0.58	0.62	0.064	0.54
	-0.059,0.011	-0.013,0.053	-0.024,0.043	-0.030,0.050	-0.065,0.0018	-0.046,0.024
Zurich	-0.021	-0.012	0.00030	0.0048	-0.021	-0.028
	(0.018)	(0.017)	(0.017)	(0.020)	(0.018)	(0.019)
	0.25	0.48	0.99	0.81	0.23	0.14
	-0.056,0.015	-0.047,0.022	-0.034,0.034	-0.035,0.045	-0.056,0.013	-0.065,0.0095
East	-0.038*	-0.011	-0.011	0.0010	-0.024	-0.029
	(0.020)	(0.019)	(0.019)	(0.023)	(0.019)	(0.020)
	0.049	0.55	0.58	0.97	0.22	0.15
	-0.077,-0.000094	-0.048,0.026	-0.048,0.027	-0.045,0.047	-0.061,0.014	-0.069,0.011
Center	-0.029	0.00095	0.00040	-0.010	-0.017	-0.016
	(0.020)	(0.019)	(0.019)	(0.023)	(0.019)	(0.020)
	0.14	0.96	0.98	0.66	0.37	0.42
	-0.068,0.0093	-0.036,0.038	-0.037,0.037	-0.055,0.035	-0.054,0.020	-0.055,0.023
Ticino	-0.030	-0.057*	-0.0056	0.037	-0.075*	-0.062+
	(0.031)	(0.029)	(0.030)	(0.038)	(0.029)	(0.035)
	0.33	0.045	0.85	0.32	0.010	0.075
	-0.091,0.030	-0.11,-0.0014	-0.064,0.053	-0.037,0.11	-0.13,-0.018	-0.13,0.0062
Couple	0.0083*	0.012***	0.0080*	0.011*	0.0062+	0.011**
	(0.0036)	(0.0035)	(0.0037)	(0.0044)	(0.0035)	(0.0037)
	0.022	0.00082	0.030	0.013	0.075	0.0040
	0.0012,0.015	0.0048,0.018	0.00080,0.015	0.0023,0.019	-0.00062,0.013	0.0034,0.018
No children (ref. n. children in HH)	0	0	0	0	0	0

One child	0.0054+<br>(0.0031)<br>0.078<br>-0.00060,0.011	0.0067*<br>(0.0029)<br>0.023<br>0.00091,0.012	0.0079*<br>(0.0031)<br>0.011<br>0.0018,0.014	0.0055<br>(0.0035)<br>0.11<br>-0.0013,0.012	0.012***<br>(0.0029)<br>0.000079<br>0.0059,0.017	0.0073*<br>(0.0031)<br>0.018<br>0.0012,0.013
Two children	0.012***<br>(0.0034)<br>0.00048<br>0.0052,0.018	0.0086**<br>(0.0032)<br>0.0076<br>0.0023,0.015	0.0098**<br>(0.0035)<br>0.0044<br>0.0031,0.017	0.012**<br>(0.0039)<br>0.0019<br>0.0045,0.020	0.0096**<br>(0.0032)<br>0.0030<br>0.0033,0.016	0.0074*<br>(0.0034)<br>0.029<br>0.00075,0.014
Three children or more	0.012*<br>(0.0049)<br>0.015<br>0.0023,0.022	0.0082+<br>(0.0047)<br>0.081<br>-0.00100,0.017	0.0077<br>(0.0050)<br>0.13<br>-0.0022,0.018	0.0092<br>(0.0058)<br>0.11<br>-0.0022,0.021	0.0084+<br>(0.0047)<br>0.073<br>-0.00078,0.018	0.0079<br>(0.0050)<br>0.11<br>-0.0018,0.018
Full-time (ref. work status)	0	0	0	0	0	0
Part-time	-0.000041<br>(0.0034)<br>0.99<br>-0.0066,0.0066	0.0027<br>(0.0033)<br>0.41<br>-0.0037,0.0091	0.00042<br>(0.0035)<br>0.90<br>-0.0064,0.0072	-0.0041<br>(0.0040)<br>0.32<br>-0.012,0.0039	-0.0011<br>(0.0033)<br>0.74<br>-0.0076,0.0054	0.000012<br>(0.0035)<br>1.00<br>-0.0069,0.0069
Unemployed	0.0052<br>(0.0076)<br>0.49<br>-0.0097,0.020	-0.00079<br>(0.0072)<br>0.91<br>-0.015,0.013	-0.0041<br>(0.0076)<br>0.59<br>-0.019,0.011	0.00019<br>(0.016)<br>0.99<br>-0.031,0.031	-0.0013<br>(0.0071)<br>0.85<br>-0.015,0.013	-0.00096<br>(0.0075)<br>0.90<br>-0.016,0.014
Studying	-0.0094<br>(0.0085)<br>0.27<br>-0.026,0.0074	-0.0060<br>(0.0080)<br>0.45<br>-0.022,0.0097	-0.0059<br>(0.0080)<br>0.46<br>-0.022,0.0097	-0.016<br>(0.010)<br>0.13<br>-0.036,0.0044	-0.0092<br>(0.0081)<br>0.26<br>-0.025,0.0067	-0.0098<br>(0.0080)<br>0.22<br>-0.025,0.0059
Non-working	-0.0062+<br>(0.0036)<br>0.083<br>-0.013,0.00081	-0.0073*<br>(0.0035)<br>0.034<br>-0.014,-0.00056	-0.0100**<br>(0.0037)<br>0.0069<br>-0.017,-0.0027	-0.0073<br>(0.0066)<br>0.27<br>-0.020,0.0057	-0.010**<br>(0.0034)<br>0.0033<br>-0.017,-0.0034	-0.011**<br>(0.0037)<br>0.0021<br>-0.019,-0.0041
1999 (ref.)	0	0	0	0	0	0
2000	-0.0051<br>(0.0035)<br>0.14<br>-0.012,0.0017	-0.0054<br>(0.0034)<br>0.11<br>-0.012,0.0012	-0.0027<br>(0.0035)<br>0.44<br>-0.0095,0.0041	-0.0075+<br>(0.0045)<br>0.094<br>-0.016,0.0013	-0.0037<br>(0.0033)<br>0.27<br>-0.010,0.0028	0.00034<br>(0.0035)<br>0.92<br>-0.0065,0.0072
2001	-0.0042<br>(0.0036)<br>0.25<br>-0.011,0.0030	-0.0020<br>(0.0035)<br>0.57<br>-0.0088,0.0049	-0.0040<br>(0.0036)<br>0.27<br>-0.011,0.0031	-0.0014<br>(0.0047)<br>0.76<br>-0.011,0.0077	0.0032<br>(0.0034)<br>0.35<br>-0.0035,0.0100	-0.00062<br>(0.0037)<br>0.87<br>-0.0079,0.0066
2002	-0.0042<br>(0.0039)<br>0.28<br>-0.012,0.0035	-0.0020<br>(0.0038)<br>0.59<br>-0.0094,0.0053	-0.0082*<br>(0.0039)<br>0.035<br>-0.016,-0.00056	-0.00069<br>(0.0049)<br>0.89<br>-0.010,0.0089	0.0014<br>(0.0037)<br>0.71<br>-0.0058,0.0086	-0.0018<br>(0.0040)<br>0.65<br>-0.0098,0.0061
2003	-0.011**<br>(0.0041)<br>0.0076<br>-0.019,-0.0029	-0.0061<br>(0.0039)<br>0.12<br>-0.014,0.0015	-0.0062<br>(0.0041)<br>0.13<br>-0.014,0.0019	-0.011*<br>(0.0050)<br>0.035<br>-0.020,-0.00076	-0.00070<br>(0.0038)<br>0.86<br>-0.0082,0.0068	-0.0026<br>(0.0043)<br>0.55<br>-0.011,0.0059
2004	-0.0070+<br>(0.0036)<br>0.051<br>-0.014,0.000022	-0.0047<br>(0.0035)<br>0.18<br>-0.012,0.0021	-0.0047<br>(0.0036)<br>0.20<br>-0.012,0.0024	-0.0098*<br>(0.0045)<br>0.028<br>-0.019,-0.0011	0.00037<br>(0.0034)<br>0.91<br>-0.0064,0.0071	0.00081<br>(0.0038)<br>0.83<br>-0.0065,0.0082
2005	-0.0018<br>(0.0038)<br>0.63<br>-0.0093,0.0056	-0.0010<br>(0.0037)<br>0.78<br>-0.0082,0.0062	-0.0015<br>(0.0038)<br>0.69<br>-0.0090,0.0059	-0.0073<br>(0.0046)<br>0.11<br>-0.016,0.0018	-0.00027<br>(0.0036)<br>0.94<br>-0.0074,0.0068	0.0019<br>(0.0040)<br>0.64<br>-0.0060,0.0098
2006	-0.0060<br>(0.0038)<br>0.12<br>-0.013,0.0015	-0.0063+<br>(0.0037)<br>0.089<br>-0.013,0.00094	-0.0049<br>(0.0038)<br>0.20<br>-0.012,0.0026	-0.0086+<br>(0.0048)<br>0.073<br>-0.018,0.00081	-0.0041<br>(0.0036)<br>0.26<br>-0.011,0.0030	-0.0028<br>(0.0041)<br>0.49<br>-0.011,0.0051
2007	0.0015<br>(0.0038)<br>0.70<br>-0.0059,0.0089	0.0023<br>(0.0036)<br>0.53<br>-0.0048,0.0094	0.0043<br>(0.0038)<br>0.26<br>-0.0031,0.012	-0.0087+<br>(0.0046)<br>0.058<br>-0.018,0.00030	0.0022<br>(0.0035)<br>0.52<br>-0.0047,0.0091	0.0028<br>(0.0040)<br>0.48<br>-0.0050,0.011
2008	-0.0030<br>(0.0038)<br>0.43<br>-0.010,0.0045	0.0017<br>(0.0037)<br>0.64<br>-0.0055,0.0089	0.0032<br>(0.0038)<br>0.41<br>-0.0043,0.011	-0.0041<br>(0.0046)<br>0.37<br>-0.013,0.0049	-0.000027<br>(0.0036)<br>0.99<br>-0.0070,0.0070	0.0033<br>(0.0041)<br>0.42<br>-0.0047,0.011
2009	-0.00050<br>(0.0038)<br>0.90<br>-0.0079,0.0069	-0.00056<br>(0.0036)<br>0.88<br>-0.0077,0.0066	0.0041<br>(0.0038)<br>0.29<br>-0.0034,0.012	-0.0077+<br>(0.0046)<br>0.091<br>-0.017,0.0012	0.0020<br>(0.0035)<br>0.57<br>-0.0049,0.0089	0.0037<br>(0.0040)<br>0.36<br>-0.0042,0.012

2011	-0.0032	-0.0021	0.00012	-0.0040	-0.0038	-0.00047
	(0.0032)	(0.0031)	(0.0034)	(0.0042)	(0.0031)	(0.0032)
	0.32	0.50	0.97	0.34	0.22	0.88
	-0.0096,0.0031	-0.0082,0.0040	-0.0065,0.0067	-0.012,0.0043	-0.0099,0.0023	-0.0068,0.0058
2014	-0.0056+	-0.0053+	-0.0039	-0.0071+	-0.0054+	-0.0048
	(0.0029)	(0.0028)	(0.0031)	(0.0039)	(0.0028)	(0.0030)
	0.059	0.062	0.20	0.066	0.057	0.11
	-0.011,0.00020	-0.011,0.00027	-0.0100,0.0021	-0.015,0.00047	-0.011,0.00016	-0.011,0.0010
2017	-0.0062*	-0.0048	-0.0025	-0.0059	-0.0050+	-0.0031
	(0.0031)	(0.0030)	(0.0032)	(0.0040)	(0.0030)	(0.0031)
	0.044	0.11	0.43	0.14	0.091	0.31
	-0.012,-0.00018	-0.011,0.0010	-0.0088,0.0037	-0.014,0.0020	-0.011,0.00080	-0.0092,0.0029
2020	-0.0062*	-0.0016	-0.0022	-0.0050	-0.0028	-0.0029
	(0.0028)	(0.0027)	(0.0029)	(0.0037)	(0.0027)	(0.0028)
	0.025	0.56	0.43	0.18	0.29	0.29
	-0.012,-0.00077	-0.0068,0.0036	-0.0079,0.0034	-0.012,0.0022	-0.0081,0.0024	-0.0083,0.0025
Constant	-0.022	-0.024	0.0032	-0.0072	0.0040	0.0057
	(0.020)	(0.019)	(0.020)	(0.027)	(0.019)	(0.020)
	0.27	0.21	0.87	0.79	0.83	0.77
	-0.061,0.017	-0.061,0.013	-0.035,0.042	-0.060,0.046	-0.033,0.041	-0.033,0.044
Individuals	28650	28716	28007	17784	27522	27346
Observations	91754	91013	85574	47938	86332	77396

Note: significance levels: + p < 0.10. * p < 0.05. ** p < 0.01. *** p < 0.001. Source: Swiss Household Panel (SHP)

Table B5. Fixed-effects estimates of the impact of being active in different types of associations on the likelihood of performing voluntary work interacted with education level

	Sports clubs<br><br>estimate<br>standard error<br>p-value<br>95% confidence interval	Cultural associations<br><br>estimate<br>standard error<br>p-value<br>95% confidence interval	Local interest groups<br><br>estimate<br>standard error<br>p-value<br>95% confidence interval	Trade unions<br><br>estimate<br>standard error<br>p-value<br>95% confidence interval	Environmental organizations<br><br>estimate<br>standard error<br>p-value<br>95% confidence interval	Charitable associations<br><br>estimate<br>standard error<br>p-value<br>95% confidence interval
Nonmember x Primary or less (ref.)	0	0	0	0	0	0
Active	0.091***	0.13***	0.19***	0.12*	0.13**	0.27***
	(0.014)	(0.018)	(0.020)	(0.056)	(0.046)	(0.022)
	5.4e-11	1.2e-13	2.4e-21	0.036	0.0041	3.3e-35
	0.064,0.12	0.097,0.17	0.15,0.23	0.0075,0.23	0.041,0.22	0.23,0.31
Active x Secondary	0.020	-0.022	-0.064**	-0.085	-0.033	-0.076**
	(0.015)	(0.019)	(0.022)	(0.059)	(0.050)	(0.024)
	0.20	0.27	0.0030	0.15	0.51	0.0015
	-0.010,0.050	-0.060,0.017	-0.11,-0.022	-0.20,0.031	-0.13,0.064	-0.12,-0.029
Active x Higher	-0.0044	-0.030	-0.099***	-0.14*	-0.091+	-0.080**
	(0.018)	(0.022)	(0.024)	(0.062)	(0.054)	(0.027)
	0.80	0.16	0.000031	0.026	0.091	0.0033
	-0.039,0.030	-0.073,0.012	-0.15,-0.052	-0.26,-0.017	-0.20,0.014	-0.13,-0.027
Age	0.019***	0.018***	0.016***	0.015***	0.017***	0.017***
	(0.0013)	(0.0013)	(0.0014)	(0.0027)	(0.0014)	(0.0014)
	7.5e-49	6.6e-40	1.6e-30	0.000000015	9.3e-35	3.9e-31
	0.017,0.022	0.015,0.020	0.013,0.019	0.010,0.021	0.014,0.020	0.014,0.019
Age * Age	-0.00020***	-0.00019***	-0.00018***	-0.00017***	-0.00019***	-0.00018***
	(0.000012)	(0.000012)	(0.000012)	(0.000029)	(0.000012)	(0.000013)
	1.1e-67	6.1e-57	4.4e-47	2.8e-09	2.1e-55	2.6e-45
	-0.00023,-0.00018	-0.00021,-0.00017	-0.00020,-0.00016	-0.00023,-0.00012	-0.00022,-0.00017	-0.00021,-0.00016
Foreigner	0.018	0.0079	-0.0015	0.022	0.0065	0.025
	(0.025)	(0.025)	(0.026)	(0.036)	(0.025)	(0.027)
	0.46	0.75	0.95	0.54	0.80	0.35
	-0.031,0.067	-0.042,0.057	-0.053,0.050	-0.048,0.092	-0.043,0.056	-0.028,0.077
Lake Geneva (ref. region)	0	0	0	0	0	0
Middleland	-0.0037	0.00059	0.0053	0.069	0.016	0.061+
	(0.033)	(0.034)	(0.033)	(0.047)	(0.037)	(0.037)
	0.91	0.99	0.87	0.14	0.67	0.096
	-0.068,0.061	-0.066,0.068	-0.060,0.071	-0.023,0.16	-0.056,0.087	-0.011,0.13
North-west	0.045	0.043	0.034	0.062	0.073	0.090+
	(0.043)	(0.043)	(0.043)	(0.060)	(0.046)	(0.047)
	0.29	0.32	0.43	0.30	0.12	0.055
	-0.039,0.13	-0.042,0.13	-0.050,0.12	-0.055,0.18	-0.018,0.16	-0.0019,0.18
Zurich	-0.037	-0.056	-0.078+	-0.054	-0.058	-0.025
	(0.044)	(0.046)	(0.044)	(0.061)	(0.049)	(0.051)
	0.40	0.22	0.078	0.37	0.24	0.62
	-0.12,0.049	-0.15,0.033	-0.16,0.0088	-0.17,0.064	-0.15,0.038	-0.12,0.075
East	-0.051	0.0080	-0.039	0.086	0.010	0.060
	(0.047)	(0.048)	(0.048)	(0.067)	(0.053)	(0.053)
	0.28	0.87	0.41	0.20	0.85	0.26
	-0.14,0.041	-0.086,0.10	-0.13,0.054	-0.045,0.22	-0.093,0.11	-0.044,0.16
Center	-0.045	-0.037	-0.077	0.022	-0.0017	0.039
	(0.047)	(0.048)	(0.048)	(0.066)	(0.051)	(0.052)
	0.34	0.44	0.11	0.74	0.97	0.46
	-0.14,0.047	-0.13,0.057	-0.17,0.016	-0.11,0.15	-0.10,0.099	-0.064,0.14
Ticino	0.013	-0.0027	-0.041	-0.031	0.077	0.066
	(0.073)	(0.072)	(0.074)	(0.12)	(0.080)	(0.087)
	0.86	0.97	0.58	0.79	0.33	0.45
	-0.13,0.16	-0.14,0.14	-0.19,0.10	-0.26,0.20	-0.079,0.23	-0.10,0.24
Couple	-0.010	-0.0050	-0.015+	-0.018	-0.0032	-0.010
	(0.0082)	(0.0084)	(0.0088)	(0.012)	(0.0089)	(0.0092)
	0.22	0.56	0.087	0.12	0.72	0.27
	-0.026,0.0061	-0.021,0.012	-0.032,0.0022	-0.042,0.0047	-0.021,0.014	-0.028,0.0079
No children (ref. n. children in HH)	0	0	0	0	0	0
One child	0.0095	0.013+	0.0029	0.0043	0.0055	0.0099

	(0.0070)	(0.0072)	(0.0075)	(0.0094)	(0.0076)	(0.0078)
	0.18	0.075	0.69	0.65	0.46	0.20
	-0.0043,0.023	-0.0013,0.027	-0.012,0.018	-0.014,0.023	-0.0093,0.020	-0.0054,0.025
Two children	0.029***	0.026***	0.020*	0.036***	0.025**	0.030***
	(0.0076)	(0.0077)	(0.0081)	(0.010)	(0.0082)	(0.0084)
	0.00016	0.00073	0.013	0.00058	0.0022	0.00037
	0.014,0.043	0.011,0.041	0.0042,0.036	0.015,0.056	0.0090,0.041	0.013,0.046
Three children or more	0.033**	0.035**	0.0085	0.027+	0.015	0.023+
	(0.011)	(0.011)	(0.012)	(0.015)	(0.012)	(0.012)
	0.0021	0.0019	0.47	0.079	0.21	0.058
	0.012,0.055	0.013,0.056	-0.014,0.031	-0.0031,0.057	-0.0083,0.037	-0.00073,0.047
Full-time (ref. work status)	0	0	0	0	0	0
Part-time	0.015+	0.0090	0.0073	0.018+	0.018*	0.0038
	(0.0077)	(0.0079)	(0.0083)	(0.011)	(0.0085)	(0.0087)
	0.053	0.26	0.38	0.099	0.037	0.66
	-0.00021,0.030	-0.0066,0.025	-0.0090,0.024	-0.0034,0.040	0.0011,0.034	-0.013,0.021
Unemployed	0.0058	-0.0075	-0.00061	-0.051	0.0043	-0.019
	(0.019)	(0.019)	(0.019)	(0.049)	(0.020)	(0.020)
	0.75	0.69	0.98	0.30	0.83	0.35
	-0.031,0.042	-0.044,0.029	-0.039,0.037	-0.15,0.046	-0.034,0.043	-0.058,0.020
Studying	0.055**	0.061**	0.056**	0.032	0.041+	0.071***
	(0.020)	(0.020)	(0.020)	(0.028)	(0.021)	(0.021)
	0.0057	0.0027	0.0046	0.25	0.052	0.00051
	0.016,0.094	0.021,0.10	0.017,0.094	-0.022,0.087	-0.00033,0.083	0.031,0.11
Non-working	0.060***	0.065***	0.060***	0.054**	0.065***	0.051***
	(0.0081)	(0.0083)	(0.0087)	(0.018)	(0.0087)	(0.0091)
	9.0e-14	8.5e-15	5.4e-12	0.0025	1.4e-13	0.000000021
	0.044,0.076	0.048,0.081	0.043,0.077	0.019,0.088	0.047,0.082	0.033,0.069
1999 (ref.)	0	0	0	0	0	0
2000	0.075***	0.082***	0.088***	0.087***	0.094***	0.085***
	(0.0091)	(0.0094)	(0.0094)	(0.014)	(0.0096)	(0.010)
	1.3e-16	1.8e-18	6.6e-21	1.5e-10	8.6e-23	3.3e-17
	0.057,0.093	0.064,0.10	0.070,0.11	0.061,0.11	0.075,0.11	0.065,0.10
2001	0.085***	0.10***	0.10***	0.14***	0.11***	0.100***
	(0.0095)	(0.0100)	(0.0100)	(0.015)	(0.010)	(0.011)
	5.0e-19	3.9e-24	3.3e-24	2.6e-21	7.4e-29	3.3e-20
	0.066,0.10	0.082,0.12	0.082,0.12	0.11,0.17	0.093,0.13	0.079,0.12
2002	0.093***	0.085***	0.10***	0.12***	0.10***	0.095***
	(0.010)	(0.011)	(0.011)	(0.015)	(0.011)	(0.012)
	6.2e-20	8.7e-16	3.8e-23	5.6e-15	3.9e-22	7.6e-16
	0.073,0.11	0.064,0.11	0.084,0.13	0.088,0.15	0.084,0.13	0.072,0.12
2003	0.087***	0.078***	0.099***	0.10***	0.10***	0.099***
	(0.010)	(0.011)	(0.011)	(0.015)	(0.011)	(0.013)
	7.7e-17	6.9e-13	1.3e-18	1.1e-11	3.0e-20	1.1e-14
	0.067,0.11	0.057,0.100	0.077,0.12	0.073,0.13	0.082,0.13	0.074,0.12
2004	0.092***	0.097***	0.11***	0.10***	0.11***	0.10***
	(0.0091)	(0.0096)	(0.0097)	(0.013)	(0.0099)	(0.011)
	1.4e-23	4.2e-24	4.1e-29	5.3e-14	1.7e-28	3.6e-22
	0.074,0.11	0.078,0.12	0.090,0.13	0.074,0.13	0.090,0.13	0.083,0.13
2005	0.080***	0.070***	0.099***	0.097***	0.099***	0.087***
	(0.0097)	(0.010)	(0.010)	(0.014)	(0.011)	(0.012)
	1.8e-16	5.7e-12	9.8e-22	2.8e-12	3.6e-21	3.7e-14
	0.061,0.099	0.050,0.090	0.079,0.12	0.070,0.12	0.079,0.12	0.065,0.11
2006	0.080***	0.083***	0.093***	0.097***	0.11***	0.096***
	(0.0097)	(0.010)	(0.010)	(0.015)	(0.011)	(0.012)
	1.5e-16	4.2e-16	3.8e-19	2.5e-11	2.0e-24	5.3e-16
	0.061,0.100	0.063,0.10	0.072,0.11	0.069,0.13	0.087,0.13	0.073,0.12
2007	0.061***	0.072***	0.086***	0.096***	0.095***	0.081***
	(0.0097)	(0.010)	(0.010)	(0.014)	(0.010)	(0.012)
	2.3e-10	1.8e-12	5.0e-17	5.5e-12	5.6e-20	7.1e-12
	0.042,0.080	0.052,0.092	0.066,0.11	0.068,0.12	0.075,0.12	0.058,0.10
2008	0.084***	0.095***	0.11***	0.12***	0.11***	0.097***
	(0.0098)	(0.010)	(0.010)	(0.014)	(0.011)	(0.012)
	1.3e-17	3.4e-20	8.4e-26	1.1e-16	2.4e-23	1.3e-15
	0.064,0.10	0.075,0.12	0.090,0.13	0.088,0.14	0.084,0.13	0.073,0.12
2009	0.057***	0.056***	0.083***	0.096***	0.079***	0.069***
	(0.0097)	(0.010)	(0.010)	(0.014)	(0.010)	(0.012)
	3.5e-09	0.000000049	1.0e-15	7.1e-12	1.8e-14	4.5e-09
	0.038,0.076	0.036,0.076	0.063,0.10	0.068,0.12	0.059,0.099	0.046,0.092
2011	-0.00073	0.00035	0.0073	0.0055	0.0062	0.0070

	(0.0066)	(0.0067)	(0.0071)	(0.010)	(0.0070)	(0.0071)
	0.91	0.96	0.30	0.58	0.37	0.32
	-0.014,0.012	-0.013,0.013	-0.0066,0.021	-0.014,0.025	-0.0075,0.020	-0.0069,0.021
2014	0.0040	0.0064	0.010	-0.00025	0.011+	0.0059
	(0.0059)	(0.0060)	(0.0064)	(0.0091)	(0.0063)	(0.0065)
	0.50	0.29	0.11	0.98	0.095	0.36
	-0.0076,0.016	-0.0055,0.018	-0.0023,0.023	-0.018,0.018	-0.0018,0.023	-0.0068,0.019
2017	0.0023	0.0024	0.0070	-0.0093	0.0062	0.0012
	(0.0061)	(0.0062)	(0.0066)	(0.0094)	(0.0065)	(0.0066)
	0.71	0.70	0.28	0.32	0.34	0.86
	-0.0097,0.014	-0.0098,0.015	-0.0059,0.020	-0.028,0.0091	-0.0066,0.019	-0.012,0.014
2020	-0.027***	-0.027***	-0.024***	-0.035***	-0.025***	-0.025***
	(0.0055)	(0.0055)	(0.0058)	(0.0084)	(0.0058)	(0.0059)
	0.0000013	0.0000011	0.000044	0.000036	0.000012	0.000017
	-0.037,-0.016	-0.038,-0.016	-0.035,-0.012	-0.051,-0.018	-0.037,-0.014	-0.037,-0.014
Constant	-0.025	0.017	0.10*	0.079	0.047	-0.0066
	(0.046)	(0.047)	(0.047)	(0.074)	(0.050)	(0.050)
	0.58	0.72	0.028	0.28	0.34	0.89
	-0.12,0.065	-0.075,0.11	0.011,0.19	-0.066,0.22	-0.050,0.14	-0.10,0.091
Individuals	12386	12228	12048	7885	11401	11169
Observations	72820	70735	66218	37049	66468	60931

Note: significance levels: + p < 0.10. * p < 0.05. ** p < 0.01. *** p < 0.001. Source: Swiss Household Panel (SHP)

Table B6. Fixed-effects estimates of the impact of being active in different types of associations on the propensity to take part in demonstration interacted with education level

	Sports clubs	Cultural associations	Local interest groups	Trade unions	Environmental organizations	Charitable associations
	estimate<br>standard error<br>p-value<br>95% confidence interval	estimate<br>standard error<br>p-value<br>95% confidence interval	estimate<br>standard error<br>p-value<br>95% confidence interval	estimate<br>standard error<br>p-value<br>95% confidence interval	estimate<br>standard error<br>p-value<br>95% confidence interval	estimate<br>standard error<br>p-value<br>95% confidence interval
Nonmember x Primary or less (ref.)	0	0	0	0	0	0
Active	0.20	0.37*	-0.022	0.86*	0.85*	-0.27
	(0.14)	(0.16)	(0.20)	(0.40)	(0.39)	(0.21)
	0.14	0.020	0.91	0.031	0.028	0.19
	-0.066,0.46	0.058,0.68	-0.41,0.36	0.079,1.63	0.091,1.62	-0.68,0.14
Active x Secondary	-0.28+	-0.41*	0.20	-0.70	-0.66	0.50*
	(0.15)	(0.18)	(0.21)	(0.43)	(0.43)	(0.23)
	0.060	0.020	0.35	0.10	0.13	0.032
	-0.58,0.012	-0.76,-0.064	-0.22,0.62	-1.54,0.14	-1.51,0.19	0.044,0.96
Active x Higher	-0.046	-0.35	0.41	-0.28	-0.76	0.52+
	(0.19)	(0.21)	(0.25)	(0.47)	(0.52)	(0.29)
	0.81	0.10	0.10	0.55	0.14	0.073
	-0.41,0.32	-0.77,0.068	-0.081,0.90	-1.21,0.64	-1.77,0.25	-0.048,1.09
Age	-0.0047	0.017	-0.0044	-0.070*	0.0099	0.019
	(0.018)	(0.018)	(0.018)	(0.032)	(0.018)	(0.021)
	0.79	0.34	0.81	0.031	0.59	0.35
	-0.040,0.031	-0.018,0.053	-0.040,0.031	-0.13,-0.0066	-0.026,0.045	-0.021,0.060
Age * Age	-0.00014	-0.00031+	-0.00018	0.00053	-0.00027	-0.00030
	(0.00017)	(0.00017)	(0.00017)	(0.00036)	(0.00017)	(0.00020)
	0.42	0.074	0.29	0.14	0.11	0.14
	-0.00047,0.00020	-0.00065,0.000031	-0.00052,0.00016	-0.00017,0.0012	-0.00061,0.000066	-0.00070,0.000098
Foreigner	0.14	0.20	0.25	0.074	0.16	0.18
	(0.20)	(0.21)	(0.21)	(0.30)	(0.20)	(0.23)
	0.48	0.33	0.23	0.80	0.43	0.45
	-0.25,0.54	-0.20,0.60	-0.16,0.66	-0.50,0.65	-0.23,0.55	-0.28,0.64
Lake Geneva (ref. region)	0	0	0	0	0	0
Middleland	0.38	0.37	0.50+	0.55+	0.49+	0.83**
	(0.29)	(0.28)	(0.28)	(0.32)	(0.29)	(0.30)
	0.18	0.19	0.075	0.087	0.089	0.0065
	-0.17,0.94	-0.18,0.91	-0.050,1.05	-0.079,1.17	-0.075,1.06	0.23,1.42
North-west	0.099	-0.69*	-0.26	-0.39	-0.017	0.074
	(0.36)	(0.35)	(0.34)	(0.43)	(0.36)	(0.42)
	0.78	0.047	0.44	0.36	0.96	0.86
	-0.60,0.80	-1.37,-0.0086	-0.93,0.41	-1.23,0.45	-0.73,0.70	-0.76,0.91
Zurich	0.69*	0.054	0.60+	0.010	0.41	0.21
	(0.34)	(0.35)	(0.34)	(0.41)	(0.36)	(0.42)
	0.045	0.88	0.078	0.98	0.26	0.62
	0.016,1.36	-0.64,0.75	-0.067,1.27	-0.79,0.81	-0.30,1.11	-0.61,1.03
East	1.20**	0.53	0.72+	-0.27	0.66+	0.76+
	(0.37)	(0.39)	(0.37)	(0.51)	(0.40)	(0.45)
	0.0013	0.18	0.053	0.59	0.099	0.089
	0.47,1.93	-0.24,1.30	-0.0093,1.45	-1.27,0.73	-0.12,1.44	-0.12,1.65
Center	0.91*	0.047	0.40	-0.12	0.45	0.64
	(0.38)	(0.40)	(0.38)	(0.51)	(0.39)	(0.44)
	0.016	0.91	0.29	0.81	0.25	0.15
	0.17,1.65	-0.73,0.82	-0.34,1.15	-1.12,0.88	-0.32,1.22	-0.23,1.50
Ticino	0.40	-0.25	0.46	0.12	-0.14	-0.96
	(0.67)	(0.62)	(0.66)	(0.81)	(0.61)	(0.89)
	0.55	0.69	0.49	0.88	0.82	0.28
	-0.91,1.70	-1.46,0.97	-0.83,1.74	-1.46,1.70	-1.34,1.06	-2.71,0.79
Couple	-0.18*	-0.16+	-0.22**	-0.23*	-0.28***	-0.10
	(0.080)	(0.081)	(0.083)	(0.10)	(0.081)	(0.092)
	0.026	0.051	0.0075	0.029	0.00052	0.26
	-0.34,-0.022	-0.32,0.00098	-0.38,-0.059	-0.43,-0.023	-0.44,-0.12	-0.28,0.076
No children (ref. n. children in HH)	0	0	0	0	0	0

One child	0.028	0.042	0.015	0.0092	0.050	-0.018
	(0.062)	(0.063)	(0.062)	(0.079)	(0.063)	(0.070)
	0.66	0.50	0.81	0.91	0.43	0.80
	-0.093,0.15	-0.081,0.17	-0.11,0.14	-0.15,0.16	-0.074,0.17	-0.16,0.12
Two children	-0.083	-0.12	-0.13+	0.0037	-0.098	-0.17+
	(0.076)	(0.077)	(0.077)	(0.098)	(0.077)	(0.085)
	0.27	0.11	0.091	0.97	0.20	0.051
	-0.23,0.066	-0.28,0.027	-0.28,0.021	-0.19,0.20	-0.25,0.053	-0.33,0.00042
Three children or more	0.016	-0.084	-0.023	0.11	0.042	-0.013
	(0.11)	(0.11)	(0.11)	(0.14)	(0.11)	(0.12)
	0.88	0.45	0.84	0.43	0.70	0.92
	-0.20,0.23	-0.30,0.13	-0.24,0.19	-0.17,0.39	-0.17,0.26	-0.25,0.23
Full-time (ref. work status)	0	0	0	0	0	0
Part-time	0.035	-0.026	0.023	0.044	0.023	0.060
	(0.071)	(0.073)	(0.075)	(0.093)	(0.075)	(0.085)
	0.62	0.72	0.76	0.64	0.76	0.48
	-0.10,0.17	-0.17,0.12	-0.12,0.17	-0.14,0.23	-0.12,0.17	-0.11,0.23
Unemployed	0.16	0.13	0.027	0.033	0.17	0.30*
	(0.13)	(0.14)	(0.14)	(0.30)	(0.14)	(0.15)
	0.23	0.34	0.85	0.91	0.23	0.045
	-0.10,0.42	-0.14,0.39	-0.24,0.29	-0.56,0.62	-0.11,0.44	0.0067,0.60
Studying	0.25	0.29+	0.29+	0.23	0.10	0.14
	(0.17)	(0.16)	(0.15)	(0.22)	(0.17)	(0.17)
	0.13	0.077	0.059	0.30	0.55	0.41
	-0.077,0.57	-0.031,0.60	-0.011,0.59	-0.20,0.67	-0.23,0.44	-0.20,0.48
Non-working	-0.0048	-0.067	0.076	0.013	0.033	0.053
	(0.079)	(0.082)	(0.084)	(0.15)	(0.082)	(0.094)
	0.95	0.41	0.37	0.93	0.69	0.57
	-0.16,0.15	-0.23,0.094	-0.088,0.24	-0.29,0.31	-0.13,0.19	-0.13,0.24
1999 (ref.)	0	0	0	0	0	0
2000	0.038	0.045	0.046	0.080	0.093*	0.056
	(0.041)	(0.041)	(0.042)	(0.060)	(0.042)	(0.045)
	0.35	0.28	0.27	0.18	0.027	0.21
	-0.042,0.12	-0.036,0.13	-0.036,0.13	-0.037,0.20	0.011,0.17	-0.032,0.14
2001	0.030	0.048	-0.010	0.100	0.11**	0.077
	(0.042)	(0.043)	(0.043)	(0.062)	(0.043)	(0.047)
	0.47	0.26	0.81	0.11	0.0077	0.10
	-0.052,0.11	-0.036,0.13	-0.095,0.074	-0.021,0.22	0.030,0.20	-0.016,0.17
2002	0.20***	0.17***	0.19***	0.30***	0.25***	0.23***
	(0.045)	(0.046)	(0.046)	(0.064)	(0.046)	(0.052)
	0.000014	0.00022	0.000061	0.0000027	0.000000051	0.0000083
	0.11,0.29	0.079,0.26	0.095,0.28	0.17,0.42	0.16,0.34	0.13,0.34
2003	0.23***	0.29***	0.26***	0.33***	0.30***	0.32***
	(0.048)	(0.048)	(0.050)	(0.066)	(0.049)	(0.057)
	0.0000020	2.3e-09	0.00000014	0.00000064	4.7e-10	0.000000028
	0.13,0.32	0.19,0.38	0.16,0.36	0.20,0.46	0.21,0.40	0.20,0.43
2004	0.31***	0.32***	0.32***	0.32***	0.33***	0.34***
	(0.042)	(0.043)	(0.044)	(0.058)	(0.044)	(0.051)
	2.6e-13	8.3e-14	7.9e-13	0.000000027	4.6e-14	1.1e-11
	0.23,0.39	0.24,0.41	0.23,0.40	0.21,0.44	0.25,0.42	0.24,0.44
2005	0.23***	0.20***	0.25***	0.18**	0.23***	0.15**
	(0.046)	(0.047)	(0.048)	(0.062)	(0.047)	(0.055)
	0.00000077	0.000017	0.00000020	0.0039	0.0000015	0.0078
	0.14,0.32	0.11,0.30	0.15,0.34	0.058,0.30	0.13,0.32	0.039,0.26
2006	0.098*	0.042	0.071	0.11+	0.083+	0.046
	(0.048)	(0.049)	(0.049)	(0.066)	(0.049)	(0.058)
	0.040	0.38	0.15	0.088	0.090	0.42
	0.0047,0.19	-0.053,0.14	-0.025,0.17	-0.017,0.24	-0.013,0.18	-0.067,0.16
2007	-0.13*	-0.079	-0.094+	-0.11+	-0.10*	-0.12*
	(0.049)	(0.050)	(0.051)	(0.065)	(0.050)	(0.059)
	0.011	0.11	0.062	0.079	0.041	0.036
	-0.22,-0.029	-0.18,0.019	-0.19,0.0047	-0.24,0.013	-0.20,-0.0043	-0.24,-0.0080
Constant	3.68***	3.45***	3.84***	5.83***	3.21***	2.92***
	(0.53)	(0.53)	(0.52)	(0.78)	(0.53)	(0.59)
	4.3e-12	6.2e-11	2.4e-13	6.1e-14	1.4e-09	0.00000064
	2.64,4.72	2.41,4.48	2.81,4.87	4.31,7.35	2.17,4.25	1.77,4.07
Individuals	27127	27126	26332	16354	25969	25790
Observations	43552	42920	41563	23459	41138	34702

Note: significance levels: + p < 0.10. * p < 0.05. ** p < 0.01. *** p < 0.001. Source: Swiss Household Panel (SHP)

Table B7. Fixed-effects estimates of the impact of being active in different types of associations on propensity to take part in boycott interacted with education level

	Sports clubs<br><br>estimate<br>standard error<br>p-value<br>95% confidence interval	Cultural associations<br><br>estimate<br>standard error<br>p-value<br>95% confidence interval	Local interest groups<br><br>estimate<br>standard error<br>p-value<br>95% confidence interval	Trade unions<br><br>estimate<br>standard error<br>p-value<br>95% confidence interval	Environmental organizations<br><br>estimate<br>standard error<br>p-value<br>95% confidence interval	Charitable associations<br><br>estimate<br>standard error<br>p-value<br>95% confidence interval
Nonmember x Primary or less (ref.)	0	0	0	0	0	0
Active	0.048<br>(0.15)<br>0.75<br>-0.25,0.35	0.38*<br>(0.18)<br>0.039<br>0.020,0.73	0.11<br>(0.22)<br>0.60<br>-0.32,0.54	0.33<br>(0.45)<br>0.47<br>-0.55,1.20	0.36<br>(0.44)<br>0.41<br>-0.50,1.22	0.19<br>(0.24)<br>0.43<br>-0.27,0.65
Active x Secondary	-0.081<br>(0.17)<br>0.63<br>-0.42,0.25	-0.40+<br>(0.20)<br>0.050<br>-0.79,0.00058	0.030<br>(0.24)<br>0.90<br>-0.44,0.50	-0.53<br>(0.48)<br>0.28<br>-1.47,0.42	-0.27<br>(0.49)<br>0.58<br>-1.23,0.69	-0.12<br>(0.26)<br>0.66<br>-0.63,0.40
Active x Higher	0.21<br>(0.21)<br>0.33<br>-0.21,0.62	-0.21<br>(0.24)<br>0.39<br>-0.68,0.27	0.048<br>(0.28)<br>0.86<br>-0.50,0.60	-0.14<br>(0.53)<br>0.80<br>-1.18,0.91	0.029<br>(0.59)<br>0.96<br>-1.12,1.18	-0.18<br>(0.33)<br>0.57<br>-0.82,0.46
Age	0.021<br>(0.020)<br>0.31<br>-0.019,0.061	0.029<br>(0.020)<br>0.16<br>-0.011,0.069	0.025<br>(0.020)<br>0.22<br>-0.015,0.065	-0.026<br>(0.036)<br>0.47<br>-0.095,0.044	0.029<br>(0.020)<br>0.16<br>-0.011,0.069	0.044+<br>(0.023)<br>0.058<br>-0.0015,0.090
Age * Age	-0.00038*<br>(0.00019)<br>0.048<br>-0.00076,-0.0000036	-0.00045*<br>(0.00020)<br>0.022<br>-0.00084,-0.000066	-0.00044*<br>(0.00019)<br>0.022<br>-0.00082,-0.000064	0.000043<br>(0.00039)<br>0.91<br>-0.00073,0.00081	-0.00050*<br>(0.00019)<br>0.011<br>-0.00088,-0.00012	-0.00050*<br>(0.00023)<br>0.030<br>-0.00095,-0.000050
Foreigner	-0.42+<br>(0.23)<br>0.067<br>-0.87,0.029	-0.38<br>(0.23)<br>0.11<br>-0.84,0.083	-0.65**<br>(0.24)<br>0.0072<br>-1.12,-0.17	-0.59+<br>(0.33)<br>0.072<br>-1.22,0.052	-0.39+<br>(0.23)<br>0.089<br>-0.84,0.060	-0.25<br>(0.27)<br>0.34<br>-0.78,0.27
Lake Geneva (ref. region)	0	0	0	0	0	0
Middleland	0.39<br>(0.32)<br>0.22<br>-0.24,1.02	0.37<br>(0.31)<br>0.23<br>-0.23,0.98	0.23<br>(0.31)<br>0.47<br>-0.39,0.85	0.82*<br>(0.35)<br>0.020<br>0.13,1.51	0.39<br>(0.32)<br>0.23<br>-0.24,1.02	0.71*<br>(0.34)<br>0.038<br>0.039,1.38
North-west	0.62<br>(0.40)<br>0.13<br>-0.17,1.41	0.12<br>(0.39)<br>0.77<br>-0.65,0.88	0.53<br>(0.38)<br>0.16<br>-0.21,1.28	0.64<br>(0.48)<br>0.18<br>-0.29,1.58	0.65<br>(0.41)<br>0.11<br>-0.15,1.45	1.44**<br>(0.48)<br>0.0028<br>0.50,2.39
Zurich	1.49***<br>(0.38)<br>0.00010<br>0.74,2.25	1.27**<br>(0.40)<br>0.0013<br>0.50,2.05	1.03**<br>(0.38)<br>0.0068<br>0.29,1.78	1.04*<br>(0.45)<br>0.021<br>0.16,1.93	1.46***<br>(0.40)<br>0.00028<br>0.67,2.25	1.49**<br>(0.47)<br>0.0015<br>0.57,2.41
East	1.49***<br>(0.42)<br>0.00039<br>0.67,2.31	1.34**<br>(0.44)<br>0.0023<br>0.48,2.20	1.31**<br>(0.42)<br>0.0017<br>0.49,2.13	1.45**<br>(0.56)<br>0.0099<br>0.35,2.56	1.57***<br>(0.45)<br>0.00044<br>0.69,2.44	2.03***<br>(0.50)<br>0.000057<br>1.04,3.02
Center	1.15**<br>(0.42)<br>0.0063<br>0.33,1.98	0.93*<br>(0.44)<br>0.035<br>0.065,1.80	0.99*<br>(0.42)<br>0.020<br>0.16,1.82	1.06+<br>(0.56)<br>0.061<br>-0.048,2.17	1.14**<br>(0.44)<br>0.0095<br>0.28,2.01	1.95***<br>(0.50)<br>0.000085<br>0.98,2.93
Ticino	2.17**<br>(0.75)<br>0.0038<br>0.70,3.64	2.31**<br>(0.71)<br>0.0011<br>0.93,3.70	2.95***<br>(0.73)<br>0.000058<br>1.51,4.39	1.99*<br>(0.89)<br>0.025<br>0.26,3.73	2.29**<br>(0.71)<br>0.0012<br>0.90,3.67	3.43***<br>(1.00)<br>0.00060<br>1.47,5.40
Couple	0.021<br>(0.090)<br>0.81<br>-0.16,0.20	0.018<br>(0.091)<br>0.85<br>-0.16,0.20	0.10<br>(0.093)<br>0.26<br>-0.079,0.29	0.0068<br>(0.11)<br>0.95<br>-0.22,0.23	0.010<br>(0.091)<br>0.91<br>-0.17,0.19	0.025<br>(0.10)<br>0.81<br>-0.18,0.23
No children (ref. n. children in HH)	0	0	0	0	0	0

One child	0.15*	0.16*	0.15*	0.15+	0.12+	0.10
	(0.069)	(0.071)	(0.070)	(0.087)	(0.071)	(0.079)
	0.033	0.020	0.037	0.094	0.080	0.19
	0.012,0.28	0.026,0.30	0.0088,0.28	-0.025,0.32	-0.015,0.26	-0.051,0.26
Two children	-0.040	-0.12	-0.14	0.085	-0.12	-0.082
	(0.086)	(0.087)	(0.086)	(0.11)	(0.086)	(0.096)
	0.64	0.15	0.11	0.43	0.16	0.40
	-0.21,0.13	-0.29,0.045	-0.30,0.033	-0.13,0.30	-0.29,0.047	-0.27,0.11
Three children or more	0.17	0.072	0.010	0.18	0.12	0.093
	(0.12)	(0.12)	(0.12)	(0.16)	(0.12)	(0.14)
	0.18	0.56	0.94	0.25	0.34	0.50
	-0.074,0.41	-0.17,0.31	-0.23,0.25	-0.13,0.49	-0.12,0.36	-0.18,0.36
Full-time (ref. work status)	0	0	0	0	0	0
Part-time	0.057	-0.038	-0.017	-0.077	-0.059	-0.043
	(0.080)	(0.082)	(0.084)	(0.10)	(0.084)	(0.096)
	0.48	0.64	0.84	0.46	0.48	0.65
	-0.099,0.21	-0.20,0.12	-0.18,0.15	-0.28,0.13	-0.22,0.11	-0.23,0.14
Unemployed	0.12	0.031	0.089	-0.084	0.11	0.16
	(0.15)	(0.15)	(0.15)	(0.34)	(0.16)	(0.17)
	0.41	0.84	0.56	0.80	0.50	0.35
	-0.17,0.42	-0.27,0.33	-0.21,0.39	-0.74,0.58	-0.20,0.41	-0.17,0.49
Studying	0.29	0.045	0.13	-0.31	-0.095	-0.0055
	(0.19)	(0.18)	(0.17)	(0.25)	(0.19)	(0.20)
	0.12	0.81	0.43	0.21	0.62	0.98
	-0.076,0.66	-0.31,0.40	-0.20,0.47	-0.80,0.17	-0.47,0.28	-0.39,0.38
Non-working	0.085	0.069	0.13	0.0018	0.057	-0.032
	(0.089)	(0.093)	(0.094)	(0.17)	(0.093)	(0.11)
	0.34	0.46	0.17	0.99	0.54	0.77
	-0.090,0.26	-0.11,0.25	-0.056,0.31	-0.33,0.33	-0.12,0.24	-0.24,0.18
1999 (ref.)	0	0	0	0	0	0
2000	0.29***	0.29***	0.23***	0.30***	0.29***	0.34***
	(0.046)	(0.046)	(0.047)	(0.066)	(0.047)	(0.051)
	1.2e-10	3.8e-10	0.0000013	0.0000072	1.2e-09	1.6e-11
	0.21,0.38	0.20,0.38	0.14,0.32	0.17,0.43	0.19,0.38	0.24,0.44
2001	0.26***	0.31***	0.21***	0.25***	0.36***	0.40***
	(0.047)	(0.048)	(0.048)	(0.068)	(0.048)	(0.053)
	0.000000024	1.1e-10	0.000015	0.00023	9.8e-14	1.1e-13
	0.17,0.36	0.22,0.40	0.11,0.30	0.12,0.38	0.26,0.45	0.29,0.50
2002	0.36***	0.41***	0.31***	0.49***	0.39***	0.46***
	(0.051)	(0.051)	(0.052)	(0.070)	(0.052)	(0.059)
	9.1e-13	1.2e-15	2.4e-09	5.5e-12	2.4e-14	3.2e-15
	0.26,0.46	0.31,0.51	0.21,0.41	0.35,0.62	0.29,0.50	0.35,0.58
2003	0.56***	0.57***	0.52***	0.54***	0.58***	0.61***
	(0.054)	(0.054)	(0.056)	(0.073)	(0.055)	(0.064)
	1.8e-25	1.3e-25	7.2e-21	8.9e-14	5.1e-26	2.6e-21
	0.46,0.67	0.46,0.68	0.41,0.63	0.40,0.69	0.47,0.68	0.48,0.73
2004	0.51***	0.46***	0.42***	0.46***	0.40***	0.41***
	(0.048)	(0.049)	(0.050)	(0.064)	(0.049)	(0.057)
	9.4e-27	2.2e-21	1.4e-17	1.4e-12	4.6e-16	1.1e-12
	0.42,0.61	0.37,0.56	0.33,0.52	0.33,0.58	0.30,0.50	0.30,0.52
2005	0.37***	0.35***	0.33***	0.28***	0.36***	0.31***
	(0.052)	(0.053)	(0.054)	(0.069)	(0.053)	(0.062)
	7.9e-13	6.5e-11	4.7e-10	0.000050	2.5e-11	0.00000087
	0.27,0.47	0.24,0.45	0.23,0.44	0.14,0.41	0.25,0.46	0.18,0.43
2006	0.13*	0.055	0.057	0.099	0.12*	0.070
	(0.054)	(0.055)	(0.055)	(0.073)	(0.055)	(0.065)
	0.016	0.31	0.30	0.18	0.028	0.28
	0.024,0.23	-0.052,0.16	-0.051,0.17	-0.044,0.24	0.013,0.23	-0.057,0.20
2007	-0.088	-0.12*	-0.10+	-0.042	-0.14*	-0.080
	(0.055)	(0.056)	(0.057)	(0.072)	(0.056)	(0.066)
	0.11	0.031	0.075	0.56	0.012	0.22
	-0.20,0.020	-0.23,-0.011	-0.21,0.010	-0.18,0.098	-0.25,-0.031	-0.21,0.049
Constant	2.72***	2.64***	2.78***	4.12***	2.36***	1.49*
	(0.60)	(0.59)	(0.59)	(0.86)	(0.60)	(0.66)
	0.0000055	0.0000080	0.0000022	0.0000015	0.000077	0.024
	1.55,3.89	1.48,3.80	1.63,3.93	2.44,5.80	1.19,3.53	0.20,2.78
Individuals	12321	12165	11988	7840	11336	11101
Observations	43094	42461	41116	23273	40698	34306

Note: significance levels: + p < 0.10. * p < 0.05. ** p < 0.01. *** p < 0.001. Source: Swiss Household Panel (SHP)
